# EPO does not promote interaction between the erythropoietin and beta-common receptors

**DOI:** 10.1038/s41598-018-29865-x

**Published:** 2018-08-20

**Authors:** Karen S. Cheung Tung Shing, Sophie E. Broughton, Tracy L. Nero, Kevin Gillinder, Melissa D. Ilsley, Hayley Ramshaw, Angel F. Lopez, Michael D. W. Griffin, Michael W. Parker, Andrew C. Perkins, Urmi Dhagat

**Affiliations:** 1 ACRF Rational Drug Discovery Centre, St. Vincent’s Institute of Medical Research, Fitzroy, Victoria 3065 Australia; 2 Department of Biochemistry and Molecular Biology, Bio21 Molecular Science and Biotechnology Institute, University of Melbourne, Parkville, Victoria 3010 Australia; 3 Australian Centre for Blood Diseases, Monash University, 99 Commercial Road, Melbourne, Victoria 3004 Australia; 4 Mater Research, University of Queensland and Metro South Health Care, South Brisbane, Queensland 4101 Australia; 5 Centre for Cancer Biology, SA Pathology and the University of South Australia, Adelaide, South Australia 5000 Australia

**Keywords:** Membrane proteins, Cell signalling

## Abstract

A direct interaction between the erythropoietin (EPOR) and the beta-common (βc) receptors to form an Innate Repair Receptor (IRR) is controversial. On one hand, studies have shown a functional link between EPOR and βc receptor in tissue protection while others have shown no involvement of the βc receptor in tissue repair. To date there is no biophysical evidence to confirm a direct association of the two receptors either *in vitro* or *in vivo*. We investigated the existence of an interaction between the extracellular regions of EPOR and the βc receptor *in silico* and *in vitro* (either in the presence or absence of EPO or EPO-derived peptide ARA290). Although a possible interaction between EPOR and βc was suggested by our computational and genomic studies, our *in vitro* biophysical analysis demonstrates that the extracellular regions of the two receptors do not specifically associate. We also explored the involvement of the βc receptor gene (*Csf2rb*) under anaemic stress conditions and found no requirement for the βc receptor in mice. In light of these studies, we conclude that the extracellular regions of the EPOR and the βc receptor do not directly interact and that the IRR is not involved in anaemic stress.

## Introduction

**Figure 1:**
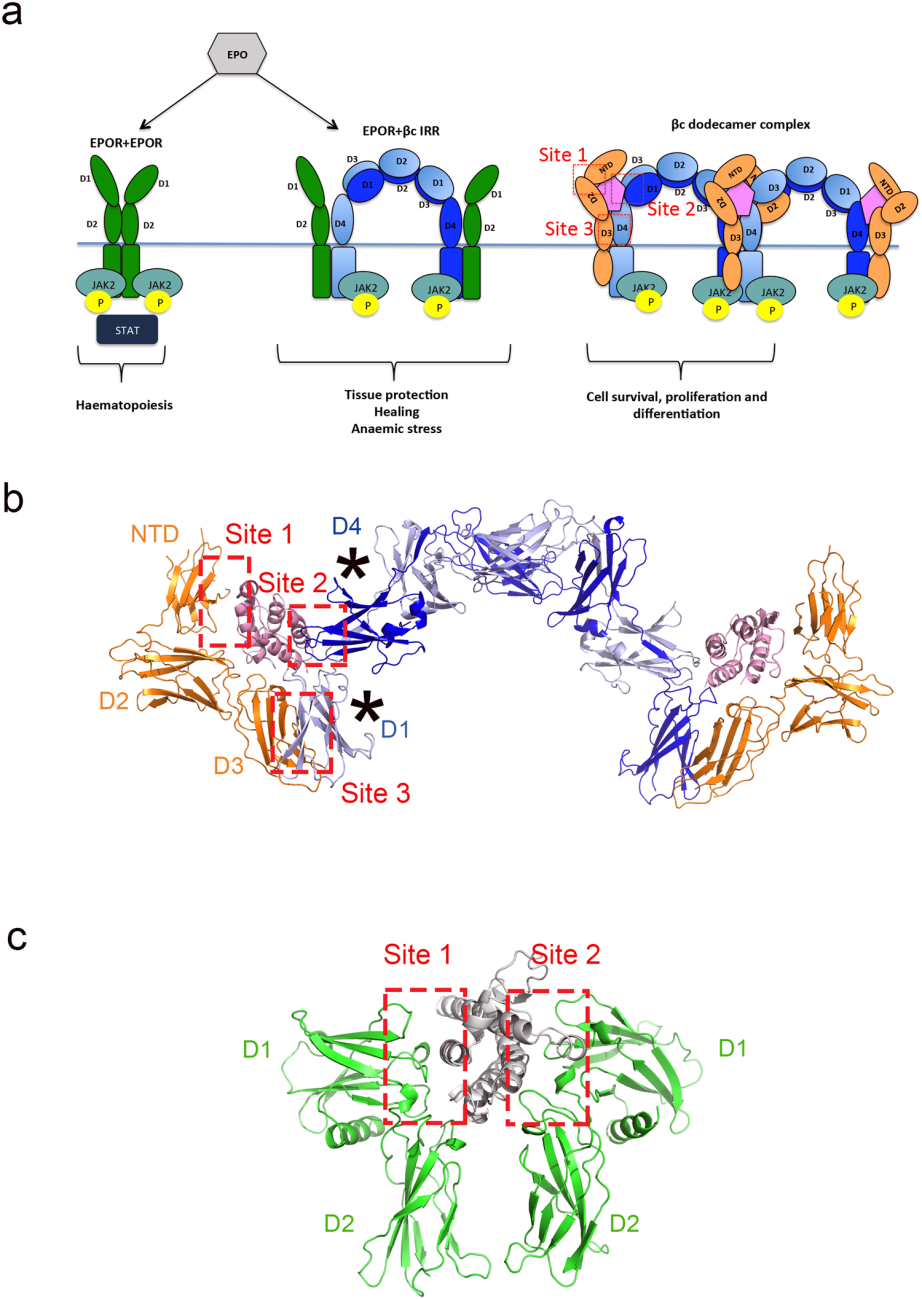
Signalling pathways triggered by EPOR homodimers, the putative EPOR:βc IRR heterodimer and the GM-CSF:GM-CSFRα:βc dodecamer complex. (**a**) EPOR (green) can form homodimers (left) or heterodimers (middle) with the βc receptor (shades of blue). Upon stimulation with EPO (grey), the EPOR homodimer promotes erythroid cell differentiation and survival. The EPOR:βc IRR heterodimer has been hypothesised to promote tissue protection and healing in non-haematopoietic cells and may play a role in anaemic stress. The GM-CSF ternary complex (GM-CSF + GM-CSFRα + βc; shades of pink, orange and blue, respectively) forms higher order signalling complexes (e.g. βc dodecamer complex) and contributes to blood cell survival, proliferation and differentiation. The different sites of interaction in the GM-CSF ternary complex (labelled as βc dodecamer complex) are indicated in red. The cell membrane location is indicated by the horizontal blue line. (**b**) The structure of the GM-CSF:GM-CSFRα:βc hexamer complex (prepared using PDB ID: 4NKQ and 4RS1)^33^ displayed in cartoon format. GM-CSF, GM-CSFRα and βc are coloured pink, orange and shades of blue respectively. For the docking studies, the βc receptor was truncated to the membrane proximal D1/D4 domains only (as indicated by *) due to the size restriction of RosettaDock. The membrane proximal D1/D4 domains are the domains in contact with the cytokine and they participate in the formation of Sites 2 and 3. (**c**) The structure of EPO:EPOR homodimer complex displayed in cartoon format (PDB ID: 1EER)^35^. The location of the EPO:EPOR interaction surfaces, Sites 1 and 2, are indicated. EPO is coloured grey and EPOR green.

Under physiological conditions, erythropoietin (EPO) is continually produced by the kidney to regulate erythropoiesis^1,2^. EPO generates erythrocytes by binding to a homodimer of the erythropoietin receptor (EPOR) on parent erythrocyte cells. The binding affinity of EPO to the EPOR homodimer expressed on erythroblasts has been reported to be ~100–200 pmol/L^3,4^. The EPOR is a single transmembrane receptor comprised of an N-terminal extracellular domain containing an EPO binding region, followed by the transmembrane domain (thought to be largely α-helical) and a C-terminal intracellular region (Fig. 1). EPO binds to EPOR on the cell surface, resulting in re-orientation and activation of the EPOR receptor^5,6^ and JAK2 phosphorylation of tyrosine (pY) residues in the intracellular region of EPOR. The phospho-tyrosine residues act as docking sites for signalling partners such as STAT5, which are then phosphorylated to mediate pathways elicited by EPOR^7–10^, culminating in the proliferation, survival and differentiation of erythrocytes.

Interestingly, EPO has also been shown to have an effect on non-haematopoietic cells such as endothelial cells, heart, kidney and cells in the central nervous system albeit at significantly higher concentrations (1–2 nmol/L) than that needed for haematopoiesis^2^. The substantial difference in affinity for the EPOR homodimers expressed by erythrocyte precursors and receptors on non-haematopoietic cells suggested that a different receptor may be involved in mediating the non-haematopoietic effects of EPO^2^. Based on immunoprecipitation studies, Brines and co-workers proposed that the non-haematopoietic effects of EPO were mediated by a heterodimer of EPOR and the beta common (βc) receptor^11–13^, which was referred to as the “Innate Repair Receptor” (IRR)^11,13–17^. The βc receptor also belongs to the type I cytokine receptor superfamily and is the main signalling subunit shared by granulocyte macrophage colony stimulating factor (GM-CSF, Fig. 1a,b), interleukin (IL)-3 and IL-5. It has been proposed that EPOR and the βc receptor are typically localised within the intracellular compartment in quiescent cells, but under stress conditions, such as hypoxia or inflammation, these receptors are rapidly mobilised to the cell surface^2,18,19^. Binding of EPO to the IRR is thought to trigger distinct signalling pathways initially mediated by JAK2 phosphorylation^2,12^, ultimately decreasing apoptosis and inflammation and promoting tissue repair^2,20,21^ (Fig. 1a).

Brines and co-workers^13^ showed that βc receptor knockout mice had less motor recovery compared to wild type mice upon EPO administration, suggesting involvement of the βc receptor in tissue repair. Furthermore, cardiomyocytes isolated from βc knockout mice were found to have higher rates of apoptosis compared to those extracted from the wild type mice after EPO treatment^13^. Blake and co-workers^12^ demonstrated that BAF-B03 cells co-expressing the EPOR and βc receptor could induce downstream JAK2 phosphorylation upon EPO stimulation. Other studies have demonstrated that the βc receptor is involved in the induction of nitric oxide upon EPO stimulation^16,22,23^. Several groups have thereafter studied EPO derivatives such as ARA290, a peptide derived from helix B of EPO^24^, that could potentially activate the proposed IRR but not the EPOR homodimer. These derivatives would help in tissue repair and protection in pathological conditions without affecting physiological haematopoiesis^24–27^, as has been demonstrated by several studies demonstrating the protective effects of these EPO derivatives^28–30^.

However, there has been no evidence of a direct interaction between the βc receptor and EPOR to date. On the contrary, studies of steady state erythropoiesis and erythroid colony formation in *Csf2rb* knockout mice (*Csf2rb*^−/−^; *Csf2rb* is the gene coding for the equivalent βc receptor in mice) have shown no involvement of the EPOR:βc IRR heteroreceptor in steady state haematopoiesis, as there was no difference in baseline haematopoiesis in βc knockout mice compared to wild type^31^. The growth of bone marrow cell cultures isolated from βc knockout and wild type mice were also similar after EPO treatment, again supporting no involvement of the βc receptor in steady state erythropoiesis^31^. Furthermore, Kanellakis and co-workers^32^ demonstrated that darbepoietin, a derivative of EPO, acted as a cardioprotective agent independently of IRR in mice when cardiac infarction was induced. The level of inflammation, fibrosis and apoptosis was reduced to a similar extent in βc knockout and wild type mice after darbepoietin administration, indicating that the βc receptor was not involved in tissue protection and repair^32^. These studies are in conflict with the proposed involvement of the EPOR:βc IRR heteroreceptor in mediating tissue protective effects in response to EPO and its derivatives.

To date, many studies have investigated the tissue protective effects of EPO and its derivatives in different models^13,25,28–30^, however no direct interaction between the EPOR and βc receptor has been reported. The crystal structures of the extracellular domains of EPOR in complex with EPO and the GM-CSF:GM-CSFRα:βc ternary complex have been determined (GM-CSFRα is the cytokine specific receptor α-subunit which interacts with the shared receptor subunit βc, Fig. 1b,c). These structures have provided molecular insight into the mechanism of assembly and activation of the EPO and GM-CSF receptor systems^5,33–35^. Previous studies have demonstrated that the extracellular regions of the type I cytokine receptors play a significant role in homo and heteroreceptor assembly *in vitro*^36^. Since the molecular determinants driving the formation of the IRR heteroreceptor have not been clearly established, we decided to investigate whether the extracellular regions of the two receptors could associate with each other *in vitro*, in the presence and absence of EPO or the helix B peptide, ARA290. We investigated the possibility of an association between EPOR and the βc receptor through *in silico* studies, *in vitro* biophysical assays and an *in vivo* mouse model (under anaemic stress). In the absence of EPO, our *in silico* docking experiments suggested that the extracellular membrane proximal domains of EPOR and the βc receptor can directly interact to produce a pre-formed IRR heteroreceptor. However, the docking studies also suggested that the extracellular membrane proximal domains of these two receptors cannot directly interact in the presence of EPO. Rather, the modelled EPO:EPOR:βc complex is structurally similar to that observed in the EPO:EPOR homodimer crystal structures (PDB IDs: 1CN4 and 1EER)^35^. Thus, our modelling data suggests that the IRR heteroreceptor is structurally possible. We next established that EPO induced rapid upregulation of the *Csf2rb* gene in an erythroid cell line via pSTAT5 binding to the promoter. In contrast, there was no involvement of the βc receptor in recovery from anaemic stress in an *in vivo* mouse model. Moreover, we could not detect any physical interaction between the extracellular domains of the two receptors *in vitro* using a variety of biophysical techniques. This is the first study to clearly demonstrate that the two receptors do not directly interact via their extracellular domains *in vitro* and do not functionally interact *in vivo* in response to anaemic stress.

## Results and Discussion

### The βc gene is a direct and immediate transcriptional target of pSTAT5

**Figure 2:**
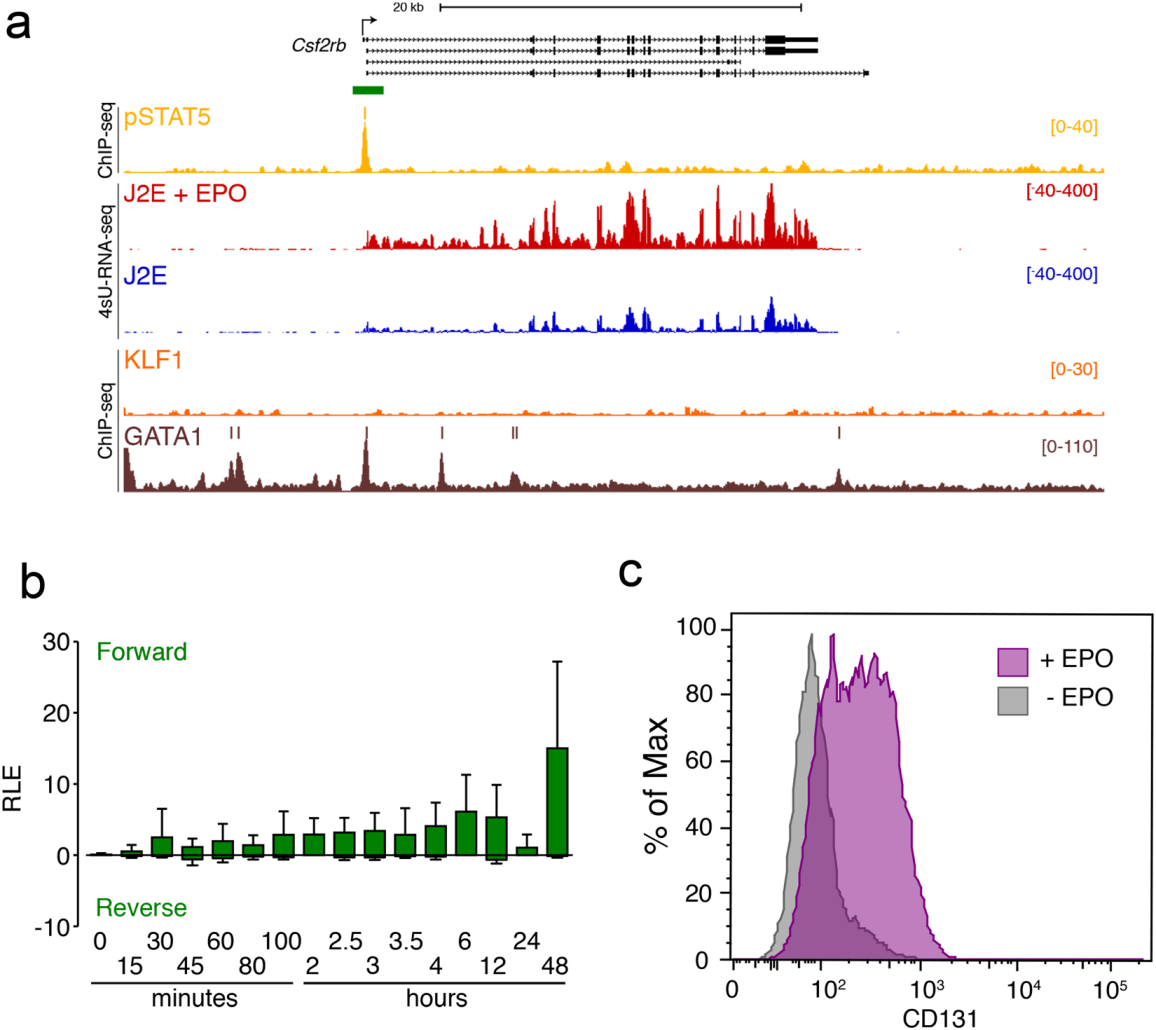
*Csf2rb* is a direct immediate transcriptional target of pSTAT5. (**a**) ChIP-seq and 4sU-RNA-seq in J2E erythroid cells following 30 min of EPO stimulation at the *Csf2rb* gene demonstrate direct immediate regulation by pSTAT5. Read density per million mapped reads (y-axis) for pSTAT5 (ochre), KLF1 (orange) and GATA1 (brown) ChIP-seq. Coloured bars above each track represent peak summits as called by MACS2. Read density profiles (y-axis) for 4sU-RNA-seq from J2E cells (blue) and J2E cells post EPO-induction (red) are displayed for both forward (+ve values) and reverse (−ve values) strands. A schematic of gene structure and alternative transcripts from RefSeq is shown in black with scale bar. (**b**) CAGE tag counts expressed as relative log expression (RLE) over the indicated pSTAT5-occupied promoter (green bar in panel A) from 0 to 24 hr post EPO stimulation. (**c**) FACS plot for CD131 (βc receptor) expression on the surface of J2E cells in the absence (grey) and 6 hr post (purple) stimulation with EPO (5 U/mL). One representative plot of 3 independent experiments is shown.

We recently undertook a study to find direct targets of pSTAT5 following stimulation of murine erythroid cells (J2E cells) by EPO^37^. We found rapid induction of *Csf2rb* (Fig. 2a) and *Csf2rb2* (not shown), and strong EPO-induced binding of pSTAT5 to the *Csf2rb* promoter. The critical erythroid transcription factor, GATA1, also binds the *Csf2rb* promoter in erythroid cell lines (Fig. 2a). Genes encoding the well-established partners of the βc receptor were not expressed in J2E cells or induced by EPO; i.e. IL-3Rα, GM-CSFRα and IL-5Rα. We confirmed EPO induction of *Csf2rb* in an independent dynamic CAGE dataset^38^ (Fig. 2b), and up regulation of the βc receptor (CD131) at the cell surface in J2E cells 6 hr after EPO stimulation (Fig. 2c). Thus, we hypothesised that an EPO-induced (“stressed”) heterodimer between EPOR and the βc receptor (or IRR), might occur in erythroid cells and provide an alternative signal generation mechanism or amplified JAK2 signalling in erythroid progenitors after stress. Such an EPOR:βc IRR heteroreceptor has been suggested to mediate survival and repair signals in response to EPO in non-haematopoietic cells^2,11–13,25,39^, but there have never been any direct biophysical or structural studies to confirm or refute such a possibility.

### *In silico* docking studies suggest that the formation of an IRR heteroreceptor is plausible

Using existing crystal structures of the EPOR and βc receptor, we constructed models of the proposed EPOR:βc IRR heteroreceptor to investigate how the extracellular regions of these two receptors might interact. We considered two possibilities, one where the IRR heteroreceptor would pre-form in the absence of EPO (i.e. resulting in the formation of Site 3, Fig. 1) and a second where EPO would mediate the heterodimerisation between EPOR and the βc receptor (i.e. with possible formation of Sites 1–3, Fig. 1). Due to the homodimeric nature of the βc receptor, the modelling presented here could involve both D4 regions of the βc homodimer (as illustrated in Fig. 1a,b).

In the absence of EPO, blind rigid body docking failed to produce an IRR heteroreceptor model where the membrane proximal domains of EPOR and the βc receptor (D2 of the EPOR and D4 of βc) were in contact forming the Site 3 interface. In a parallel approach, we constrained the D2 domain of EPOR and the D4 domain of βc to be membrane proximal throughout the docking process, facilitating the formation of Site 3 (i.e. biased docking using RosettaDock)^40–42^. For each EPOR crystal structure chain used in the biased docking (i.e. PDB IDs: 1CN4 A chain, 1ERN B chain and 1EER B chain), the top ten ranked IRR heteroreceptor models were retained for analysis. Hence thirty IRR heteroreceptor models in total were analysed and, for each EPOR crystal structure chain, the IRR heteroreceptor model with the largest Site 3 interface was selected as a representative (Models 2–4, Supplementary Fig. 1b–d). A summary of the Site 3 interactions for IRR Models 2–4 is given in Supplementary Tables S1 and S2.

**Figure 3:**
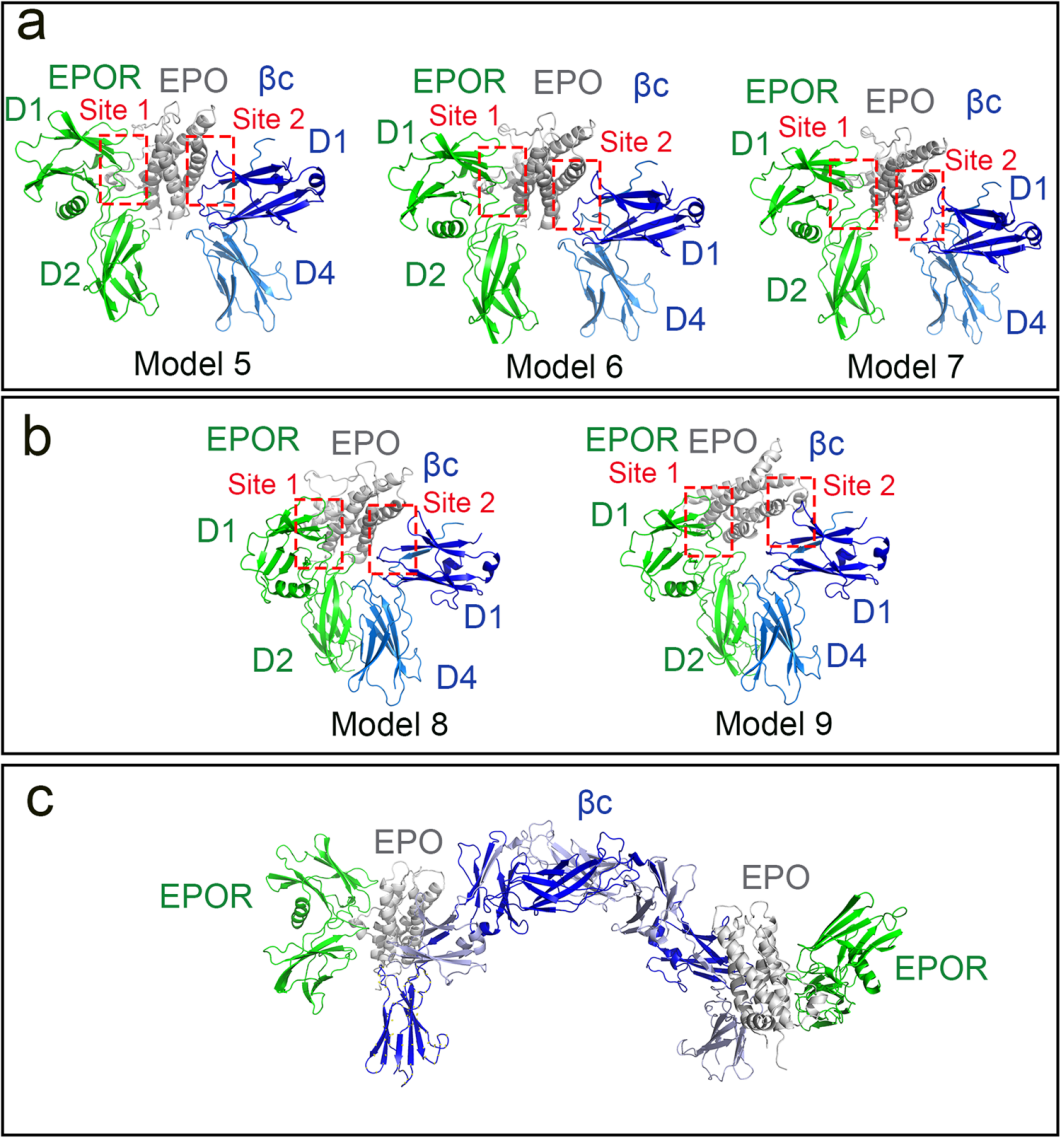
Docking the extracellular domains of EPOR and the βc receptor in the presence of EPO. EPO:EPOR: βc IRR heteroreceptor models arising from the biased docking approach. EPO is shown as a grey cartoon, EPOR in green and βc (D1/D4) in blue. (**a**) Models 5–7 show EPO interacting with EPOR via Site 1 and βc (D1/D4 only) via Site 2. When EPO is bound to Sites 1 and 2, the membrane proximal domains of EPOR (D2) and βc (D4) are too far apart to interact and form Site 3. These models are structurally analogous to the EPO:EPOR homodimer complex. (**b**) To construct Models 8–9, EPO was docked to Site 1 of EPOR in the pre-formed heteroreceptor Models 2 and 3 (i.e. Site 3 has already formed, Supplementary Fig. S1b,c). When the EPOR: βc interaction at Site 3 is maintained, EPO is physically unable to interact at Site 2. (**c**) The βc receptor is a homodimer (Fig. 1b) and therefore EPO and/or EPOR (model 5 used as representative) could interact at one or both ends of the βc receptor homodimer.

In the presence of EPO, two different scenarios were investigated while modelling the IRR heteroreceptor. In the first scenario, EPO was assumed to bind to the EPOR:βc IRR heteroreceptor in a similar manner to that observed in the crystal structures of the EPO:EPOR complex (PDB IDs: 1CN4, 1EEN), i.e. EPO interacting at Sites 1 and 2, and no formation of Site 3 (Figs 1c and 3a). The top twenty ranked biased docking solutions were analysed and the three EPO:EPOR:βc IRR models with the largest Site 1 and 2 interfaces were selected as representative (Models 5–7, Fig. 3a, Supplementary Tables S3–S6). When EPO is bound to the EPOR:βc IRR heteroreceptor in this manner, the membrane proximal domains of the two receptors cannot physically interact at Site 3 (Fig. 3a). In the second scenario, EPO was docked into either Site 1 of EPOR or Site 2 of the βc receptor in the pre-formed EPOR:βc IRR heteroreceptor Models 2–4 (i.e. Site 3 formed, Supplementary Fig. S1b–d). Docking EPO to EPOR Site 1 in Models 2 and 3 resulted in minor steric clashes between EPO and the βc receptor at Site 2, whereas there were major Site 2 steric clashes in Model 4. The two crude EPO:EPOR:βc complexes were used as input to RosettaDock in an attempt to relieve the minor steric clashes between EPO and βc, however this was unsuccessful and in each case EPO was ejected from Site 2 (Models 8–9, Fig. 3b, Supplementary Tables S3–S6). When EPO helix A is docked into Site 2 of the βc receptor, in an analogous manner to the way in which GM-CSF interacts with βc at Site 2 (Fig. 1), EPO cannot physically fit into the groove formed by EPOR and βc while the Site 3 interaction between EPOR and βc is maintained.

Based on our docking studies, and protein-protein interface analysis, it appears that the only feasible way EPO can interact with the EPOR:βc IRR heteroreceptor is via scenario one, i.e. formation of Sites 1 and 2, no Site 3 (Models 5–7, Fig. 3a). This is structurally analogous to the EPO:EPOR complexes (PDB IDs: 1CN4, 1EEN), however since βc is a homodimer the IRR heteroreceptor may have the stoichiometry 2 EPO: 2 EPOR:1 βc homodimer (Fig. 3c). Although our modelling studies suggest that the extracellular regions of EPOR and the βc receptor could interact in the absence of EPO (i.e. formation of Site 3), the two receptors would be required to undergo structural rearrangement to enable the binding of EPO (i.e. formation of Sites 1 and 2) and subsequent receptor activation. In light of these results, we investigated the possibility of an interaction between the extracellular domains of EPOR and the βc receptor in the absence, and presence, of EPO or the ARA290 peptide *in vitro*, and built up a model that would aid us in understanding the mechanism of formation of the IRR and the association of EPO to the IRR.

### The extracellular regions of EPOR and the βc receptor do not associate in absence of EPO

We initially carried out binding experiments using commercially acquired EPOR (Sino Biological, China) and our in-house purified βc receptor. The commercially available EPOR protein demonstrated clear binding to the βc receptor in microscale thermophoresis (MST) and surface plasmon resonance (SPR) assays (Supplementary Fig. S2a,b), but not in analytical size exclusion chromatography (SEC) and in pull down assays. This prompted us to investigate the quality of the commercially acquired protein sample using analytical ultracentrifugation (AUC), which demonstrated that the commercially acquired protein was aggregated and thus unsuitable for our studies. Hence, an in-house EPOR protein was purified using established protocols^43^ and verified to be appropriate for carrying out binding studies (see Supplementary Fig. S2c).

Several studies have implicated the association of EPOR with the βc receptor to form an IRR heteroreceptor in response to high levels of EPO^2,11,13–18^, but there is no published biophysical evidence of their interaction *in vitro*. Thus, the in-house purified EPOR and βc receptor were used to investigate whether the extracellular domains of these two receptors interact in the presence or absence of EPO. The identity of the proteins was confirmed by visualisation in anti-His Western blotting and by tryptic digest mass spectrometry analysis. Circular dichroism indicated proper folding of EPOR (Supplementary Fig. S3), predicting the protein to consist of 2% α-helices, 50% β-strands, 8% turns and 37% disordered, which was consistent with the DSSP secondary structure analysis^44^ of the EPOR homodimer structure (PDB ID: 1ERN). Analysis of the βc receptor indicated that it contained 11% α-helices, 35% β-strands, 24% turns and 31% disordered, which was consistent with the DSSP secondary structure analysis^44^ of the βc homodimer structure (PDB ID: 2GYS)^45^. The proper folding of the proteins has been tested in positive control binding assays (see below) to ensure the functionality of the proteins.

**Figure 4:**
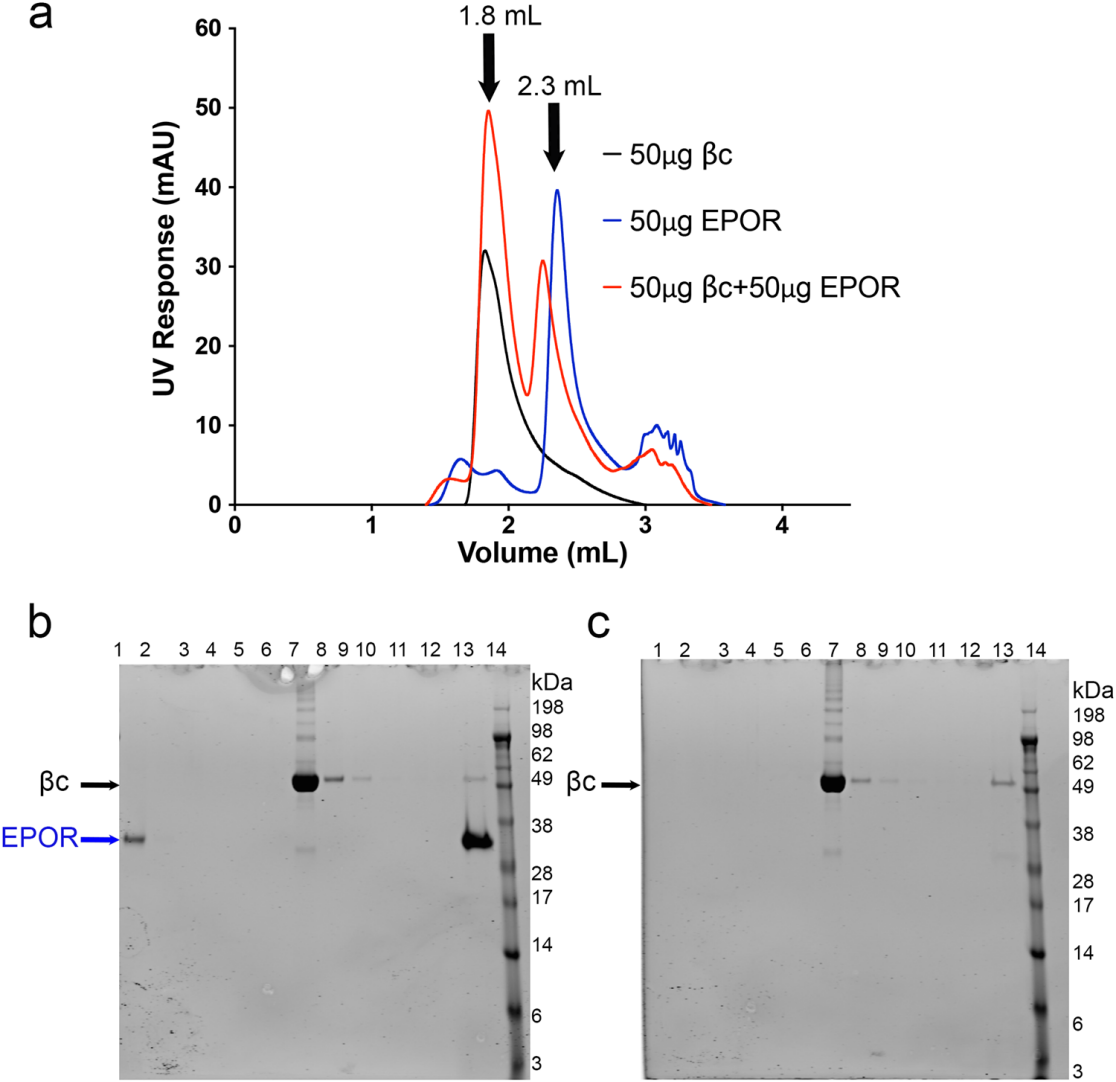
Analytical SEC and pull down assays could not detect binding between the purified EPOR and βc receptor proteins. (**a**) Overlay of the 280 nm UV absorbance spectra for EPOR, the βc receptor and EPOR + βc injected into the SEC column. (**b**) Pull down assays were carried out with EPOR-His incubated with His-Tag resin and then with the βc receptor after washing. (**c**) A control experiment with initial incubation with buffer instead of EPOR-His was also carried out. In both panels (b) and (c), samples loaded on the coomassie stained gels were 1: Supernatant after incubation with resin, 2–6: Washes 1–5 after initial incubation with resin, 7: Supernatant after incubation with βc, 8–12: Washes 6–10 after incubation with βc, 13: Resin after washing, 14: Molecular weight marker. EPOR-His and the βc receptor are indicated by the blue and black arrows, respectively. Shown are representative data from a single experiment. N = 3 separate experiments were carried out.

Initially, we looked for evidence of the formation of the EPOR:βc heterodimer in solution using analytical SEC and pull-down assays. Previous SEC studies have successfully shown the formation of higher molecular weight complexes between GM-CSF, GM-CSFRα and βc, thereby demonstrating that high affinity complexes involving the βc receptor can be detected using this biophysical technique^34^. For our analytical SEC studies, EPOR and the βc receptor were first injected separately and then together (in molar ratios 1:1 and 1:3), after incubation for one hr on ice. The EPOR:βc mixture was analysed in two different running buffers at different pH values (pH 4.5 and pH 7), and with different incubation times (1–4 hr on ice) but in all cases, two separate peaks were obtained in the elution profiles when the EPOR + βc mixtures were injected (see Supplementary Fig. S4). One peak corresponded to the βc receptor at ~1.8 mL and the second peak to EPOR at ~2.3 mL (Fig. 4a). The fractions collected from the SEC were visualised on SDS-PAGE gels and analysed by mass spectrometry to confirm the results. The absence of an elution volume peak <1.8 mL indicates that there was no higher molecular weight complex formed between EPOR and the βc receptor.

In a different approach, a pull down assay was conducted, where the resin was first incubated with EPOR-His, washed thoroughly with buffer and then incubated with the βc receptor. The resin was then washed and the bound proteins were eluted. EPOR-His was eluted from the resin (band at 32 kDa), but there was no band on the gel corresponding to the βc receptor (50 kDa), indicating that βc did not bind to EPOR-His (Fig. 4b). Although there was a faint band at the βc receptor molecular weight in the elution, this same faint band was also observed in the control experiment, indicating some residual non-specific binding of βc to the resin. Thus, the analytical SEC and pull down assays suggest that either the extracellular domains of EPOR and the βc receptor do not interact with each other or that the interaction may be too weak to be detected by these methods. Therefore, more sensitive techniques such as SPR and MST were pursued to investigate the possibility of IRR formation.

**Figure 5:**
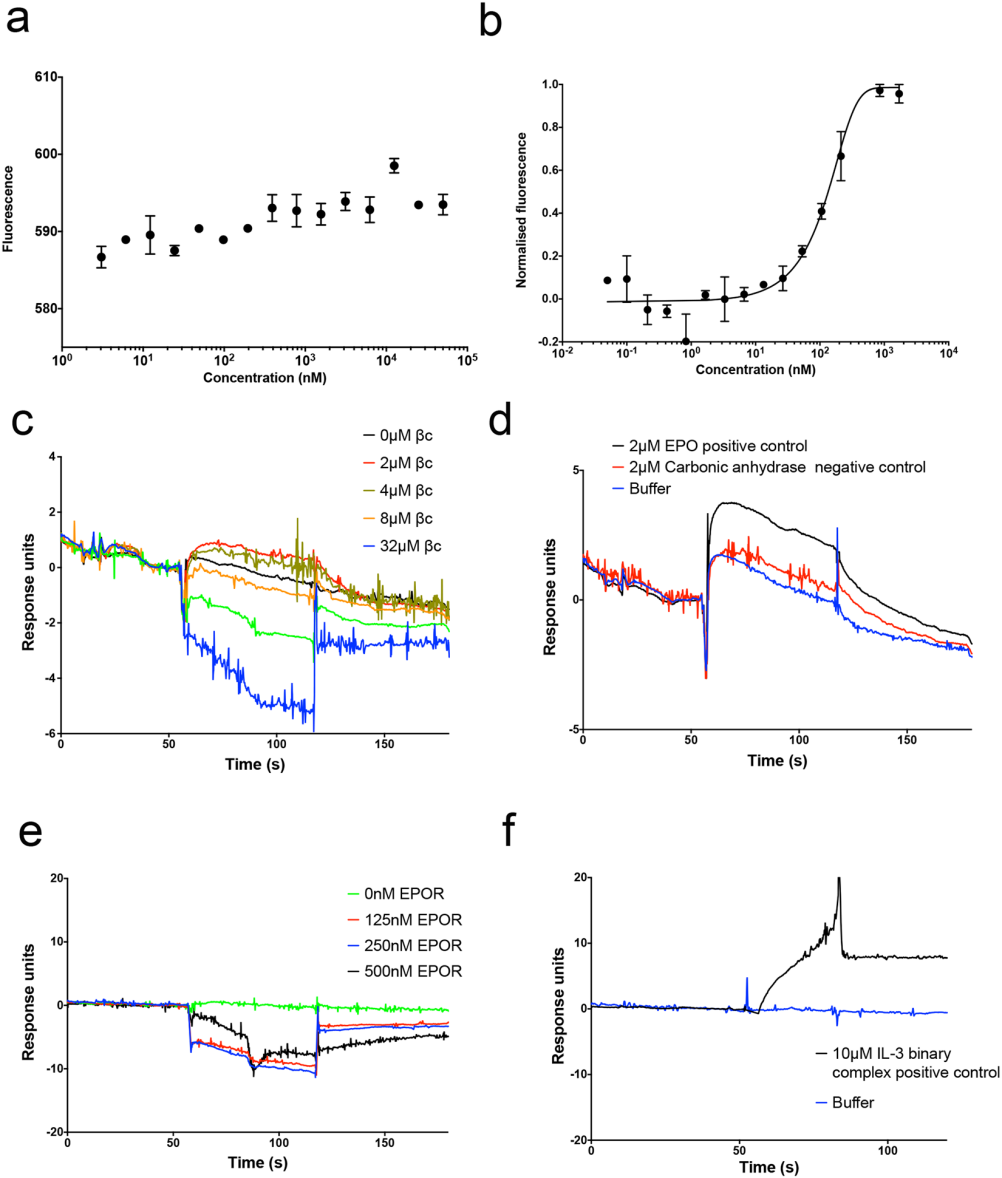
MST and SPR data suggest that the purified extracellular domains of EPOR and the βc receptor do not associate. (**a**) The purified EPOR (30 nM) was labelled and titrated with a range of βc receptor (1.5–50000 nM) concentrations in MST assays with laser powers of 60%. No binding curve was observed between EPOR and the βc receptor. (**b**) A control experiment with a concentration range of unlabelled EPOR (0.05–1700 nM) titrated against the labelled EPOR (30 nM) was carried out at 60% laser power and indicated formation of the EPOR homodimer with a K_d_ of 166 ± 16 nM (mean ± SEM). (**c**) No binding was observed when different concentrations of the βc receptor were injected onto a surface of immobilised EPOR in SPR assays. (**d**) The integrity of the chip surface in the SPR assays was validated by injecting EPO as a positive control and carbonic anhydrase as a negative control. (**e**) Likewise, no binding was observed when different concentrations of EPOR were injected onto a surface of immobilised βc receptor in SPR experiments. (**f**) The integrity of the chip surface of immobilised βc receptor in SPR was validated by injecting the IL-3 binary complex (IL-3 + IL-3Rα) as a positive control. Shown are representative data from a single SPR experiment. Each MST and SPR experiment was conducted in duplicate, with N = 3 separate experiments.

For the MST experiments, purified EPOR (30 nM) was labelled and titrated against different concentrations of unlabelled βc receptor (1.5–50000 nM) The mixture was excited with different infra-red laser powers (20%, 40%, 60%) and the thermophoretic experiment was repeated several times using different glass capillaries (standard, hydrophobic, hydrophilic), but none of these experiments exhibited a conclusive binding event (Fig. 5a). However, when labelled EPOR was titrated against unlabelled EPOR (0.05–1700 nM) as a positive control, a well-defined binding curve corresponding to the formation of an EPOR homodimer was observed (Fig. 5b). This is the first report of an affinity for the extracellular domains of the EPOR homodimer *in vitro* in the absence of EPO (K_d_ of 166 ± 16 nM), which was also observed in the apo EPOR homodimer structure^5^. The data are consistent with previous studies, which showed that the extracellular domains of EPOR were sufficient for the formation of the homodimer in cell lines expressing truncated mutants of EPOR lacking the intracellular domain^46^.

In parallel, we performed SPR experiments where biotinylated EPOR was immobilised on a CM5 chip through an anti-biotin antibody (Fig. 5c,d). The surface showed binding of EPO as a positive control, but no binding to carbonic anhydrase as a negative control, validating the chip surface strategy (Fig. 5d). The βc receptor (0–32 μM) was injected over the chip surface, but no binding was observed (Fig. 5c). The experiment was repeated several times using different buffers, however there was no evidence of βc binding. We also tested binding of different concentrations of EPOR (0–500 nM) to biotinylated βc receptor immobilised on the chip surface but again no binding was observed (Fig. 5e). As a positive control, we demonstrated binding of the IL-3 binary complex (IL-3 + IL-3Rα) to the βc receptor confirming that the membrane proximal domains (D4) of the βc homodimer were available for interaction and that the βc surface was active (Fig. 5f). Both the MST and SPR experiments indicate that extracellular regions of EPOR and the βc receptor do not associate to form the IRR heteroreceptor.

### EPO does not bring the extracellular domains of EPOR and the βc receptor together

**Figure 6:**
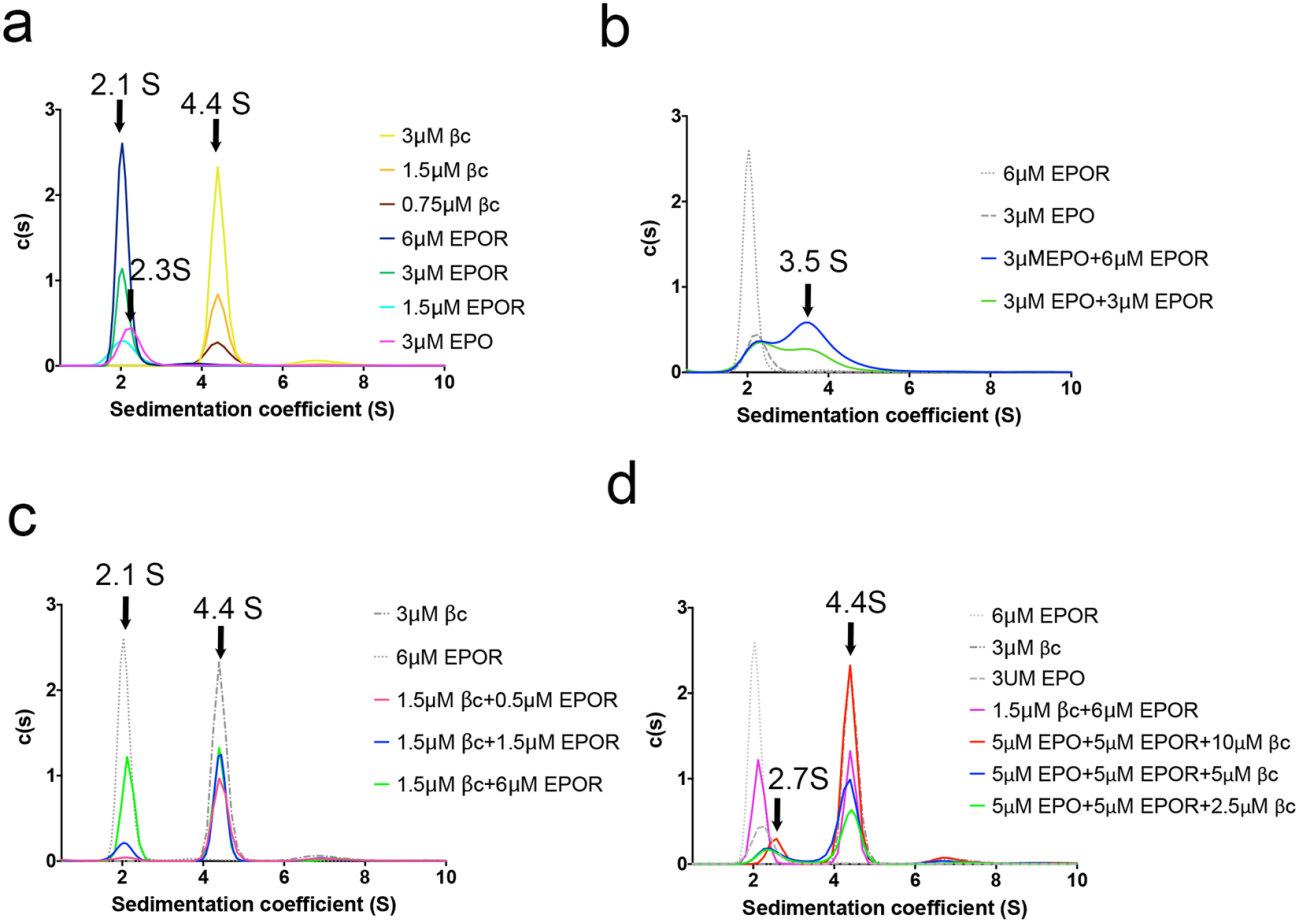
AUC studies indicate no association between the EPOR and βc receptor extracellular domains. (**a**) The EPOR, EPO and βc receptor proteins were characterised by AUC studies with sedimentation coefficients of 2.1 S, 2.3 S and 4.4 S, respectively. (**b**) EPO bound to the EPOR in the AUC studies, forming a complex with a sedimentation coefficient of approximately 3.5S. (**c**) Binding of the βc receptor to EPOR in the absence of EPO was not detected. (**d**) Binding of the βc receptor to EPOR in the presence of EPO was not detected. Shown are representative data from a single experiment.

Analytical ultracentrifugation (AUC) studies were undertaken to investigate whether EPO played a role in bringing the two receptors together. AUC is an in-solution technique with no modification of the protein, and thus mitigates the effects of protein modification that occurs to some extent in MST (dye labelling) and SPR (biotinylation and immobilisation of protein) experiments. We found EPOR and βc do not interact in the AUC system. We then included EPO in our experiments, to determine whether the ligand was required to mediate the interaction between EPOR and the βc receptor. Sedimentation coefficients of 2.1S, 2.3S and 4.4S were determined for the EPOR monomer, EPO and βc homodimer, respectively (Fig. 6a). Different combinations of the proteins were then evaluated for possible interaction. We demonstrated binding of EPO to EPOR, with a sedimentation coefficient of 3.5S (Fig. 6b). However, we could not identify any interaction between EPOR and the βc receptor in the absence (Fig. 6c) or presence (Fig. 6d) of EPO. When all three proteins were present, the signal for EPO and the EPO + EPOR complex was relatively low. As a result, the peaks corresponding to EPO and the EPO + EPOR complex were apparent as a single peak with average sedimentation coefficient of approximately 2.7S, and significant broadening of this peak was observed at the lower βc concentrations. Overall, there was no significant change in the βc receptor peaks in the mixtures assayed and no new peak at a higher sedimentation coefficient was observed, indicating that no higher order complex formation between EPOR and the βc receptor was detectable over these concentration ranges. Thus, the AUC data further confirmed that the extracellular domains of EPOR and the βc receptor do not specifically interact *in vitro*, in the absence or presence of EPO.

### ARA290 cannot mediate an interaction between the extracellular domains of EPOR and the βc receptor

**Figure 7:**
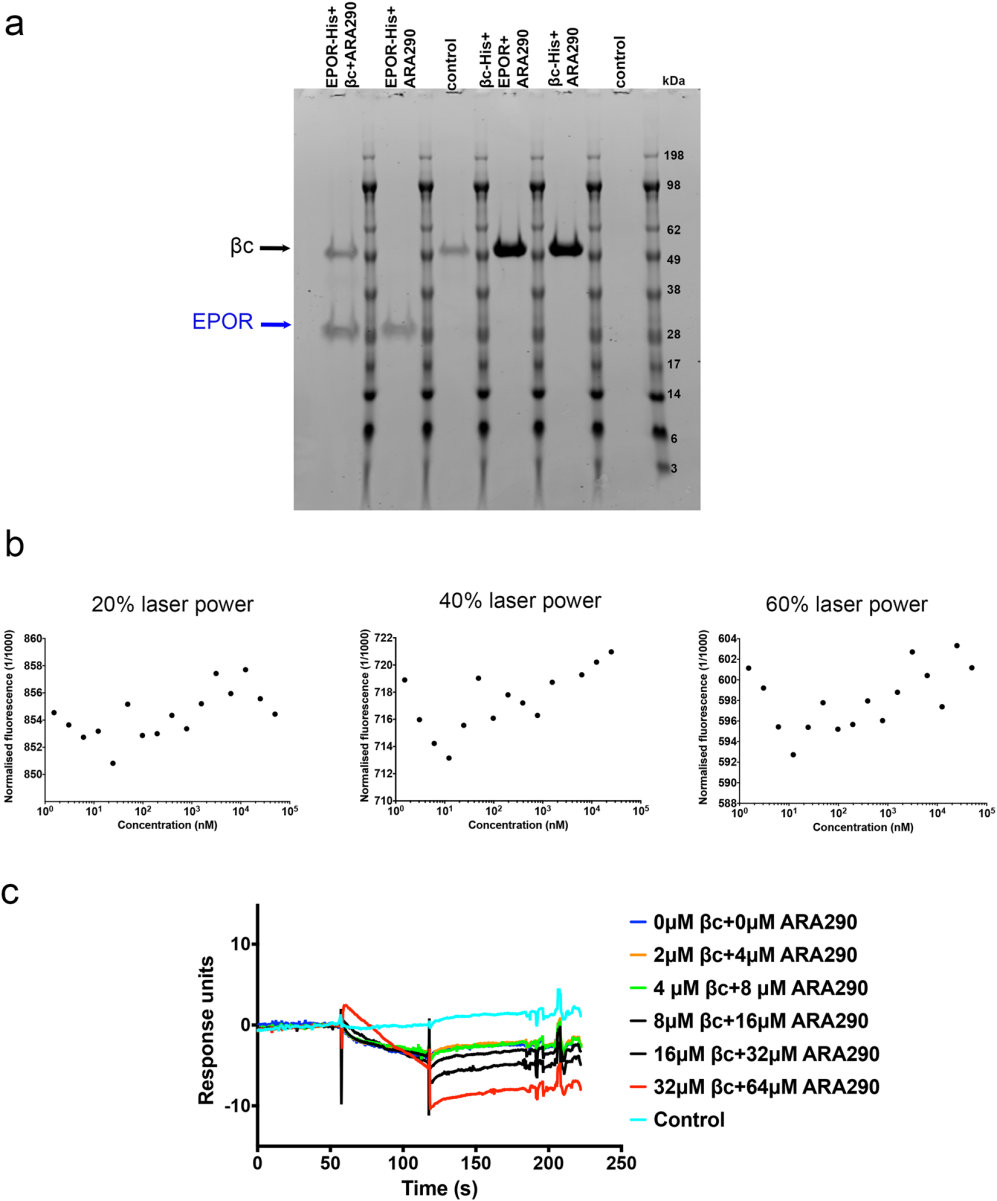
EPOR and the βc receptor do not associate in the presence of the ARA290 peptide. (**a**) Pull down assays were carried out with either EPOR-His or βc-His immobilised on resin incubated with either the βc receptor or EPOR in the presence of ARA290 after washing. In a second experiment, the immobilised EPOR-His or βc-His was incubated with the ARA290 peptide only. In control experiments, the resin was incubated with either untagged βc receptor or EPOR. Each experiment was repeated to validate pull-down data. (**b**) The effect of ARA290 upon EPOR and βc receptor association was also investigated using MST. Purified EPOR (30 nM) was labelled and incubated with ARA290 (60 nM) before being titrated with a range of βc receptor concentrations (1.5–50000 nM) at laser powers of 20%, 40% and 60%. Shown is representative data from a single experiment. Each experiment was carried out as three separate repeats (N = 3). (**c**) SPR studies with a mixture of βc (0–32 µM) and ARA390 (0–64 µM) injected over immobilised EPOR. Shown is representative data from a single experiment. Each experiment was carried out as three separate repeats (N = 3).

Lastly, we tested whether the peptide ARA290 could bring the extracellular domains of EPOR and the βc receptor together^28^. ARA290 has been reported to preferentially bind to the proposed IRR heteroreceptor and not to the EPOR homodimer^24,26–28,47^. We first carried out pull down assays where EPOR-His was immobilised on resin before being incubated with ARA290 or βc + ARA290. The components bound to the resin were eluted and visualised on an SDS-PAGE gel. No binding of EPOR to ARA290 or βc + ARA290 was observed (Fig. 7a), although some non-specific binding of the βc receptor to the resin was observed, as confirmed by the control experiment (i.e. absence of any EPOR-His on the resin). Mass spectrometry confirmed that the ARA290 peptide was not pulled down with EPOR. The reverse experiment was also performed, where βc-His was immobilised on the resin and incubated with EPOR or EPOR + ARA290. Again, no binding of the peptide or EPOR + ARA290 was observed as determined by SDS-PAGE gel and mass spectrometry. We further explored the possibility of the ARA290 peptide binding to EPOR and/or the βc receptor in MST assays. Labelled EPOR was incubated with ARA290 and a range of βc concentrations (1–50000 nM) were titrated. The MST results confirmed that the extracellular domains of EPOR and βc did not interact, even in the presence of ARA290 (Fig. 7b). SPR was also carried out to test whether the βc receptor could bind to immobilised EPOR in the presence of ARA290 (2:1 ratio of ARA290:βc) injected. The βc receptor (0–32 μM) was injected in the presence of ARA290 (0-64 μM) but no binding was observed to the immobilised EPOR (Fig. 7c), consistent with the pull-down and MST assays results.

### Recovery from anaemic stress is not compromised in the absence of *Csf2rb*

**Figure 8:**
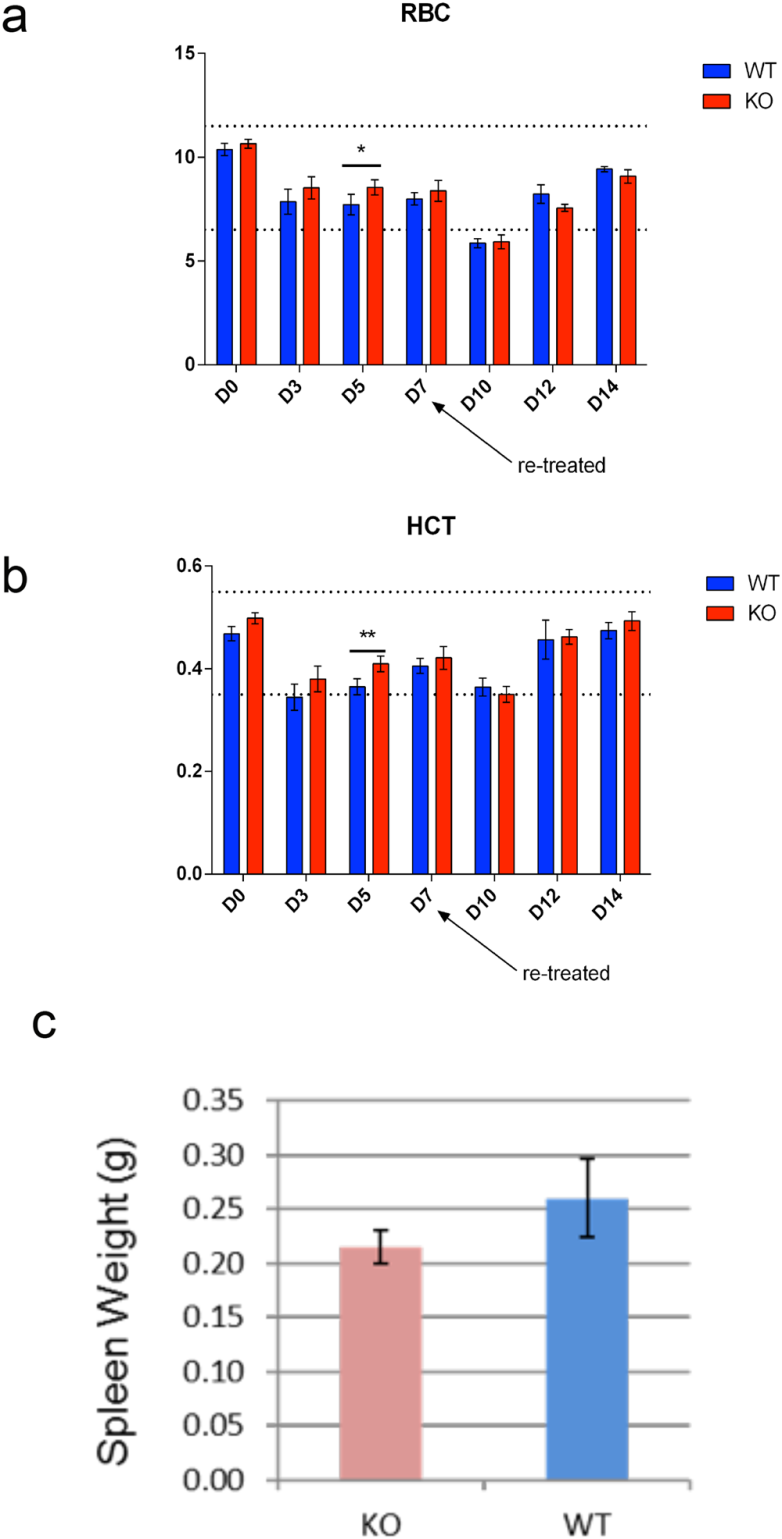
Normal recovery of *Csf2rb* knockout mice from anaemic stress. (**a**) Serial red blood cell count (RCC) in *Csf2rb* (red) and wild type (blue) after an initial (60 mg/kg) and second (90 mg/kg) injection of phenylhydrazine i.p. (**b**) Haematocrit (HCT) measurement in the same cohort. Values are mean ± SD of four mice; **p value < 0.01; *p value < 0.05 by student’s t test (**c**) Average spleen weight in the same cohort at D14 (N = 4 in each group); error bars indicate ± SD.

There are conflicting reports about the role of the βc receptor in mediating signals in erythroid progenitor cells in response to EPO. One group has reported that the forced overexpression of the βc receptor in BaF3/EPOR cells leads to increased sensitivity to EPO^11^. However, there are no reported changes in red blood cell numbers, size or haematocrit in *Csf2rb* knockout mice^31^. These analyses were undertaken without any stress placed on red blood cell production rates. Since the majority of studies have shown that the βc receptor only associates with EPOR under stress conditions, and not in steady state haematopoiesis, we investigated the possibility of the formation of the IRR heteroreceptor in response to stress in erythroid cells. Moreover, direct upregulation of *Csf2rb* in J2E cells (equivalent to BFU-e) after exposure to EPO (Fig. 2), suggested a possible role for the βc receptor in recovery from the stress of phenylhydrazine-induced haemolysis in *Csf2rb* knockout mice (see Materials and Methods). However, there were no significant differences in the recovery of red blood cell count (Fig. 8a), other red blood cell parameters (not shown) or haematocrit (Fig. 8b) in *Csf2rb*^−/−^ mice compared to wild type C57BL/6 mice after a single dose of phenylhydrazine (60 mg/kg). Furthermore, there was no difference in recovery after a second higher dose of phenylhydrazine (90 mg/kg) given on day 7 (D7, Fig. 8a) and the spleen weights were similar between *Csf2rb*^−/−^ mice and wild type mice at D14 (Fig. 8c). Therefore, we conclude that the βc receptor plays no role in EPO signalling *in vivo* at baseline or in response to anaemic stress.

## Conclusions

Previous studies have suggested that an IRR formed by the heterodimerisation of EPOR and the βc receptor might mediate EPO-induced tissue healing and repair^2,11,13–18^, and that such a heterodimer might increase JAK-STAT signalling in response to EPO in blood cell lines^11^. Our *in silico* docking studies demonstrated that the formation of a heterodimeric complex between the extracellular domains of EPOR and the βc receptor was plausible. If the IRR heteroreceptor forms in the absence of EPO, subsequent ligand binding would cause the membrane proximal domains of the two receptors to move apart and there would be little or no interaction between the extracellular domains of these receptors (i.e. no Site 3 interface, Fig. 3a,c). The resulting EPO:EPOR:βc complex would therefore be structurally analogous to what is observed in the EPO:EPOR crystal structures. However, our multi-faceted biophysical studies using the extracellular domains of the two receptors showed that the proposed IRR heteroreceptor does not form in the absence or presence of EPO or the EPO-derived peptide, ARA290, under a broad range of *in vitro* conditions (pH 4.5–8, 50–500 mM NaCl, 4–25 °C, immobilised or in solution). It is possible that the transmembrane and/or intracellular regions of the two receptors might play a role in mediating an interaction between the EPOR and the βc receptor, as suggested by previous studies^48,49^. Blake and co-workers^12^ also demonstrated that the W282R mutation in the intracellular domain of EPOR and deletion of the intracellular box 1 of βc abrogated any downstream phosphorylation of JAK2 upon EPO treatment of BAF-B03 cells co-expressing both the EPOR and βc receptor.

Moreover, our haemolytic stress studies suggest that there is no functional interaction between intact EPOR and the βc receptor. Bohr and co-workers^19^ previously suggested that the IRR heteroreceptor is formed intracellularly and is externalised upon stress through membrane rafts in response to injury or a surge of EPO levels. The spatial and temporal coordination of stress and EPO surge were not investigated in this study and may be relevant factors that encourage IRR formation or externalisation. Another possibility is that there is no direct interaction between EPOR and the βc receptor to mediate healing and tissue protection or recovery from stress. Rather, the effects of EPO might be indirect through an intermediary agent, as previously suggested by Brines and co-workers^13^, who suggested that there might be other components to the EPOR:βc protein complex. This hypothesis is consistent with several studies that identified the presence, or upregulation, of the βc receptor together with EPOR during injury or stress induced in mouse models or cells^11,13–17^, but no evidence of a direct interaction between the two receptors has ever been reported. Although no direct interaction between the extracellular regions of these two receptors was detected, it is possible that the transmembrane and/or intracellular regions of the EPOR and βc receptor may associate to form the IRR. There may well be a link between EPOR and the βc receptor in the mediation of tissue repair and protection under stress, but our present studies demonstrated that there was no direct interaction between the extracellular regions of these two receptors and that the IRR is not formed under anaemic stress.

## Materials and Methods

### ChIP-Seq and RNA-seq

ChIP-seq for pSTAT5 in murine erythroid cells and RNA-seq to find direct targets of JAK-STAT5 signalling were undertaken as recently reported^37^. The primary data from Gene Accession Omnibus under accession GSE94301 was uploaded into the UCSC Genome Browser for visualisation. The Zenbu Genome Browser was interrogated for dynamic CAGE data changes after EPO (epoetin alpha from Janssen-Cilag) stimulation of the murine erythroid cell line, J2E^38^. For FACS, J2E cells were harvested 6 hr after ± EPO (5 U/mL), and stained for surface CD131 (βc receptor) using BD Biosciences PE-conjugated rat anti-mouse CD131 (Cat#559920).

### *In silico* docking of EPOR to the βc receptor in the absence of EPO

Initial starting complexes of the membrane proximal domains of EPOR (D2, PDB IDs: 1ERN_A, 1EER_B, 1CN4_A) and the βc receptor (D1 of first monomer and D4 of second monomer, PDB ID: 4NKQ) were generated using using PyMOL v1.8.2.2^50^ and SYBYL-X 2.1.1^51^. To identify the individual EPOR and βc components used in each IRR heteroreceptor model the following nomenclature was used: [EPOR (PDB ID_chain]_[βc_GM-CSF; D1/D4 only]. The docking studies were performed using ClusPro2.0^52,53^ for a blind rigid dock and RosettaDock Suite 3.4 software^40–42^ for a biased docking. The protein interfaces, surfaces and assemblies (PISA) service at the European Bioinformatics Institute^54^ was used analyse the interacting interfaces and PredHS^55^ and KFC2^56^ web servers were used to predict hot spot residues within the protein-protein interaction interface. All docking results were visualised in PyMOL. The details for feasible pre-formed EPOR:βc IRR heteroreceptor models are tabulated in Supplementary Tables S1 and S2 and the detailed methods are described in the Supplementary methods.

### *In silico* docking of EPOR to the βc receptor in the presence of EPO

Two different scenarios were investigated for the docking studies in the presence of EPO. In the first scenario, it was assumed that EPO binds to the EPOR:βc IRR heteroreceptor in a similar manner to the way it interacts with the EPOR homodimer, i.e. with the formation of Sites 1 and 2 but not Site 3 (Fig. 1a,c). D4 of βc (D1/D4 only) from the GM-CSF ternary complex (PDB ID: 4NKQ)^33^ was aligned to D2 of EPOR in the two EPO:EPOR complexes (PDB IDs: 1EER and 1CN4)^35^. The EPO:EPOR (PDB IDs: 1EER A and B chains for EPO and EPOR, respectively and 1CN4 A and C chains for EPOR and EPO, respectively) and βc (D1/D4 only) components were merged together to form two starting EPO:EPOR:βc IRR complexes and these were used as input to RosettaDock; with the EPO:EPOR component defined as the receptor and βc (D1/D4 only) as the ligand.

In the second scenario, EPO was assumed to interact with the pre-formed IRR heteroreceptor in a similar manner to that of GM-CSF in the ternary complex, i.e. with the formation of Sites 1–3 (Fig. 1a,b). The models arising from the EPOR:βc docking in the absence of EPO were representative of this scenario and were used to dock EPO to EPOR Site 1 and, alternatively, to dock EPO to Site 2 of the βc receptor using RosettaDock. The protein interaction surfaces were analysed as described above. The details for putative EPO:EPOR:βc IRR heteroreceptor models are tabulated in Supplementary Tables S3–S6.

### Purification of the extracellular region of EPOR

The pFastbac1 plasmid encoding the extracellular domains of the EPOR, with an N-terminal secretion tag (MKFLVNVALVFMVVYISYIYAD) and a C-terminal cleavable (TEV) 6xHis tag, was commercially cloned (*BamHI* and *EcoRI* restriction Sites, Bioneer, Australia). The in-house EPOR was expressed in High Five cells and purified using nickel affinity chromatography and SEC with the identity and quality of the protein verified (described in Supplementary data).

### Purification of the extracellular region of the βc receptor

The pFastbac1 plasmid encoding the extracellular regions of the βc receptor with an N-terminal secretion tag (MKFLVNVALVFMVVYISYIYAD) and a C-terminal cleavable 6xHis tag was kindly donated by Dr. Timothy Hercus (Centre for Cancer Biology, SA Pathology and the University of South Australia, Adelaide, South Australia 5000, Australia). The βc receptor was expressed in attached *Sf*21 insect cells and purified by nickel affinity chromatography and SEC, with the identity and quality of protein verified (described in Supplementary data).

### Analytical size exclusion chromatography (SEC)

Analytical SEC was carried out using an S200 increase GL 5/150 column (GE Healthcare) (50 mM phosphate, 150 mM NaCl pH 7 or 50 mM Na acetate, 150 mM NaCl pH 4.5) at room temperature. βc protein (50 μg or 150 μg) and EPOR protein (50 μg) were first each injected separately for characterisation. 50 μg each of EPOR and βc were then mixed together and incubated for one hr on ice before injection on the SEC column. The different samples of EPOR:βc proteins were incubated and analysed. N = 3 separate experiments were carried out.

### Pull down assays

The C-terminal 6xHis tags on the purified βc-His and EPOR-His were cleaved overnight using TEV protease, with cleavage confirmed by performing an anti-His Western blot. EPOR-His (20 μg) or βc-His (20 μg) were first incubated with equilibrated complete His-tag purification resin (10 μg, Sigma Aldrich) for 1.5 hr, gentle rotation at 4 °C, followed by five washes with buffer (100 μL wash buffer, 50 mM phosphate, 300 mM NaCl, 5 mM imidazole, pH 8). The resin with immobilised protein was then incubated with βc receptor or EPOR (cleaved His tags) in the absence or presence of ARA290 (1:3 molar ratio of immobilised protein:ARA290) for 2 hr at room temperature and then again thoroughly washed. A small amount of resin was then boiled in reducing buffer to elute any bound proteins, which were resolved by SDS-PAGE. A control experiment where the resin was initially incubated with buffer instead of EPOR-His or βc-His was also carried out. Further experiments where the immobilised protein (EPOR-His or βc-His) was incubated with ARA290 were also conducted. The SDS-PAGE gels were coomassie stained for visualisation. The ARA290 (pEQLERALNSS, pE = pyroglutamic acid) was commercially manufactured by Genscript as per details in^28^. Each experiment was repeated (N = 2) to validate pull-down results.

### Microscale thermophoresis (MST) assays

The MST studies were carried out using a Nanotemper Monolith NT.115 instrument. The EPOR protein was labelled with dylight NHS Ester Dye 650 (Thermofisher Scientific) in a 1:1 molar ratio, with excess unbound dye removed by gravity flow SEC. 10 μL of the labelled EPOR at 30 nM was either directly titrated with 10 μL of the different concentrations of unlabelled protein (either βc receptor or EPOR) or was mixed with ARA290 in a 2:1 molar ratio of ARA290:EPOR and incubated for 1 hr on ice before being titrated with the unlabelled βc receptor and loaded into Monolith capillaries. All dilutions were made using the MST buffer (50 mM Tris, 10 mM MgCl_2_, 150 mM NaCl, 0.05% Tween 20, pH 7.5) provided in the Monolith capillaries kit (catalogue number MO-K002). The fluorescence of the capillary contents were analysed at 70% red light LED power to ensure standardisation of the labelled EPOR present in each capillary before each run. The contents of each capillary was then excited using an infra-red laser (20%, 40% and 60% laser power) for 30 sec, allowing thermophoresis to occur, followed by 5 sec back diffusion (laser off). The normalised fluorescence (fluorescence after thermophoresis/initial fluorescence) was then recorded for each sample concentration and the data analysed using the Monolith NT Analysis software 1.5.41. A Sigmoidal 4 parameter logistic regression, with X as log (concentration), was used to fit the analysed data using GraphPad software^57^. Each experiment was conducted with N = 3 separate experiments. For the positive control experiment, i.e. unlabelled EPOR titrated against labelled EPOR, the 60% laser power data was used to calculate a K_d_ expressed as mean ± SEM.

### Surface plasmon resonance (SPR) assays

SPR assays were performed on a Biacore T200 instrument (GE Healthcare) at 25 °C, 30 μL/sec and in 50 mm phosphate, 150 mM NaCl, 0.05% Tween, pH 7 running buffer. Both the reference and active flow cells of the CM5 chip (GE Healthcare) were activated using NHS/EDC and an anti-biotin antibody (B1750-06X) purchased from US Biological and were immobilised until saturation (8000–9000 RU) on both flow cells, followed by ethanolamine blocking, as per guidelines provided in the GE Healthcare product manual. EPOR and βc proteins were biotinylated using EZ link sulfo-NHS biotin (ThermoFisher Scientific), with excess biotin removed by SEC (S200 10/300 increase column, GE Healthcare). Biotinylated EPOR was immobilised on active flow 2 cell and biotinylated βc on active flow cell 4, aiming at an R_max_ of 100 for the level of immobilisation. All samples injected were buffer exchanged into the running buffer. Each surface was tested with a positive control (EPO for EPOR and IL-3 + IL-3 receptor α-subunit (IL-3Rα) for βc) before injection of samples of interest. All binding curves were double referenced, with blank flow cell and buffer subtractions in the analyses using the T200 Biacore Evaluation software. EPO was purchased from Genscript (Z02975-50) and IL-3 + IL-3Rα was prepared as described in^58^. Each experiment was conducted in duplicate, with N = 3 separate experiments.

### Analytical ultracentrifugation (AUC)

Sedimentation velocity AUC studies were carried out using a Beckman Coulter XL-I centrifuge equipped with UV/Vis scanning absorbance optics. Samples and reference buffer (10 mM HEPES, 500 mM NaCl pH 7.2) were loaded in double sector 12 mm cells with quartz windows and centrifuged at 20 °C and a rotor speed of 50,000 rpm (201,600 *g*) using either an An-60Ti 4-hole or An-50Ti 8-hole rotor. Radial absorbance data were recorded during sedimentation at wavelengths chosen based on the absorbance of the protein samples. Buffer density (1.01944 g/mL) and viscosity (0.01057 P) were calculated using SEDNTERP^59^. The data were analysed and fitted to a continuous sedimentation coefficient model [c(s) model] using Sedfit^60,61^, with the partial specific volume calculated from SEDNTERP^59^ (0.7207 for βc, 0.7278 for EPOR, 0.7418 for EPO, 0.7389 for IL-3, 0.7253 for IL-3Rα and an average of 0.73 for protein mixtures). EPO (3 μM), EPOR (1.5, 3, 6 μM) and βc (0.75, 1.5, 3 μM) were analysed for reference. Different combinations of samples (3 μM EPO + 3 μM EPOR, 3 μM EPO + 6 μM EPOR, 1.5 μM βc + 0.5 μM EPOR, 1.5 μM βc + 1.5 μM EPOR, 1.5 μM βc + 6 μM EPOR, 5 μM EPO + 5 μM EPOR + 2.5 μM βc, 5 μM EPO + 5 μM EPOR + 5 μM βc and 5 μM EPO + 5 μM EPOR + 10 μM βc) were then analysed. Figures were generated using either SigmaPlot version 12.0 (Systat Software, San Jose, CA) or Graphpad Prism version 7.0. EPO was purchased from Genscript (Z02975-50) for the AUC studies. Each set of experiments was performed once at the different concentrations.

### Mouse studies

Female *Csf2rb*^−/−^ mice^31^ were maintained on a C57BL/6 genetic background. Female age-matched wild type C57BL/6 mice (8–12 weeks) were used as controls. Phenylhydrazine was injected at day zero (D0) (60 mg/kg i.p.). Serial whole blood samples (20 μL) were collected prior to injection (D0), and again at D3, D5 and D7 for a complete blood count (CBC) as described^62^. At D7, a second injection of phenylhydazine (90 mg/kg i.p.) was delivered to mimic a delayed second anaemic stress event. Further CBCs were undertaken at D10, D12 and D14 when the mice were sacrificed and spleen weight/body weight ratios measured. Red blood cell count and haematocrit data are presented as mean ± SD for N = 4 mice in each group. Spleen weights (g) for the same cohort are expressed as the average ± SD. A student’s test was used (in GraphPad) to determine if differences between the two groups for each of the measured blood parameters or spleen weights was significant or not. All animal experiments were undertaken with the approval of the University of Queensland Animal Ethics Committee.

All experiments were performed in accordance with the relevant Institutional guidelines and regulations and, where required, approval was sought and granted by the relevant Institutional Biosafety Committees of St. Vincent’s Institute, University of Melbourne, Monash University and University of Queensland.

## Electronic supplementary material

Supplementary Information

## References

[1] Fisher JW (2003). Erythropoietin: physiology and pharmacology update. Exp Biol Med (Maywood).

[2] Brines M, Cerami A (2008). Erythropoietin-mediated tissue protection: reducing collateral damage from the primary injury response. J Intern Med.

[3] Broudy VC, Lin N, Brice M, Nakamoto B, Papayannopoulou T (1991). Erythropoietin receptor characteristics on primary human erythroid cells. Blood.

[4] Krzyzanski W, Wyska E (2008). Pharmacokinetics and pharmacodynamics of erythropoietin receptor in healthy volunteers. Naunyn Schmiedebergs Arch Pharmacol.

[5] Livnah O (1999). Crystallographic evidence for preformed dimers of erythropoietin receptor before ligand activation. Science.

[6] Moraga I (2015). Tuning cytokine receptor signaling by re-orienting dimer geometry with surrogate ligands. Cell.

[7] Damen JE (1997). The role of erythropoietin receptor tyrosine phosphorylation in erythropoietin-induced proliferation. Leukemia.

[8] Li K, Miller C, Hegde S, Wojchowski D (2003). Roles for an Epo receptor Tyr-343 Stat5 pathway in proliferative co-signaling with kit. J Biol Chem.

[9] Witthuhn BA (1993). JAK2 associates with the erythropoietin receptor and is tyrosine phosphorylated and activated following stimulation with erythropoietin. Cell.

[10] Menon MP (2006). Signals for stress erythropoiesis are integrated via an erythropoietin receptor-phosphotyrosine-343-Stat5 axis. J Clin Invest.

[11] Jubinsky PT, Krijanovski OI, Nathan DG, Tavernier J, Sieff CA (1997). The beta chain of the interleukin-3 receptor functionally associates with the erythropoietin receptor. Blood.

[12] Blake TJ, Jenkins BJ, D’Andrea RJ, Gonda TJ (2002). Functional cross-talk between cytokine receptors revealed by activating mutations in the extracellular domain of the beta-subunit of the GM-CSF receptor. J Leukoc Biol.

[13] Brines M (2004). Erythropoietin mediates tissue protection through an erythropoietin and common beta-subunit heteroreceptor. Proc Natl Acad Sci USA.

[14] Leist M (2004). Derivatives of erythropoietin that are tissue protective but not erythropoietic. Science.

[15] Khan AI (2013). Erythropoietin attenuates cardiac dysfunction in experimental sepsis in mice via activation of the beta-common receptor. Dis Model Mech.

[16] Su KH (2011). beta Common receptor integrates the erythropoietin signaling in activation of endothelial nitric oxide synthase. J Cell Physiol.

[17] Coldewey SM (2013). Erythropoietin attenuates acute kidney dysfunction in murine experimental sepsis by activation of the beta-common receptor. Kidney Int.

[18] Collino M, Thiemermann C, Cerami A, Brines M (2015). Flipping the molecular switch for innate protection and repair of tissues: Long-lasting effects of a non-erythropoietic small peptide engineered from erythropoietin. Pharmacol Ther.

[19] Bohr S (2015). Modulation of cellular stress response via the erythropoietin/CD131 heteroreceptor complex in mouse mesenchymal-derived cells. J Mol Med (Berl).

[20] Nairz M (2017). Cibinetide dampens innate immune cell functions thus ameliorating the course of experimental colitis. Sci Rep.

[21] Kebschull, L. *et al*. EPOR2/betacR2-independendent effects of low-dose epoetin-alpha in porcine liver transplantation. *Biosci Rep* **37**, 10.1042/BSR20171007 (2017). 10.1042/BSR20171007 PMC5715127 29127105

[22] Sautina L (2010). Induction of nitric oxide by erythropoietin is mediated by the {beta} common receptor and requires interaction with VEGF receptor 2. Blood.

[23] Su KH (2012). AMP-activated protein kinase mediates erythropoietin-induced activation of endothelial nitric oxide synthase. J Cell Physiol.

[24] Swartjes M (2014). ARA 290, a peptide derived from the tertiary structure of erythropoietin, produces long-term relief of neuropathic pain coupled with suppression of the spinal microglia response. Mol Pain.

[25] Brines M (2015). ARA 290, a nonerythropoietic peptide engineered from erythropoietin, improves metabolic control and neuropathic symptoms in patients with type 2 diabetes. Mol Med.

[26] van Velzen M (2014). ARA 290 for treatment of small fiber neuropathy in sarcoidosis. Expert Opin Investig Drugs.

[27] Hache G (2016). ARA290, a Specific Agonist of Erythropoietin/CD131 Heteroreceptor, Improves Circulating Endothelial Progenitors’ Angiogenic Potential and Homing Ability. Shock.

[28] Niesters M (2013). The erythropoietin analog ARA 290 for treatment of sarcoidosis-induced chronic neuropathic pain. Expert Opinion on Orphan Drugs.

[29] van Rijt WG (2013). ARA290, a non-erythropoietic EPO derivative, attenuates renal ischemia/reperfusion injury. J Transl Med.

[30] Masaaki Wanatabe, Y. s., *et al* and Makiko Kumagai-Braesch. An Engineered Innate Repair Receptor Agonist, ARA 290, Protects Rat Islets from Cytokineinduced Apoptosis. *J Diabetes Metab* **7**, 10.4172/2155-6156.1000708 (2016).

[31] Scott CL (2000). Reassessment of interactions between hematopoietic receptors using common beta-chain and interleukin-3-specific receptor beta-chain-null cells: no evidence of functional interactions with receptors for erythropoietin, granulocyte colony-stimulating factor, or stem cell factor. Blood.

[32] Kanellakis P (2010). Darbepoetin-mediated cardioprotection after myocardial infarction involves multiple mechanisms independent of erythropoietin receptor-common beta-chain heteroreceptor. Br J Pharmacol.

[33] Broughton SE (2016). Conformational Changes in the GM-CSF Receptor Suggest a Molecular Mechanism for Affinity Conversion and Receptor Signaling. Structure.

[34] Hansen G (2008). The structure of the GM-CSF receptor complex reveals a distinct mode of cytokine receptor activation. Cell.

[35] Syed RS (1998). Efficiency of signalling through cytokine receptors depends critically on receptor orientation. Nature.

[36] Waters MJ, Brooks AJ, Chhabra Y (2014). A new mechanism for growth hormone receptor activation of JAK2, and implications for related cytokine receptors. JAKSTAT.

[37] Gillinder KR (2017). Direct targets of pSTAT5 signalling in erythropoiesis. PLoS One.

[38] Arner E (2015). Transcribed enhancers lead waves of coordinated transcription in transitioning mammalian cells. Science.

[39] Brines M, Cerami A (2012). The receptor that tames the innate immune response. Mol Med.

[40] Lyskov S (2013). Serverification of molecular modeling applications: the Rosetta Online Server that Includes Everyone (ROSIE). PLoS One.

[41] Chaudhury S (2011). Benchmarking and analysis of protein docking performance in Rosetta v3.2. PLoS One.

[42] Lyskov S, Gray JJ (2008). The RosettaDock server for local protein-protein docking. Nucleic Acids Res.

[43] Gustin SE (2001). Expression, crystallization and derivatization of the complete extracellular domain of the beta(c) subunit of the human IL-5, IL-3 and GM-CSF receptors. Eur J Biochem.

[44] Touw WG (2015). A series of PDB-related databanks for everyday needs. Nucleic Acids Res.

[45] Carr PD, Conlan F, Ford S, Ollis DL, Young IG (2006). An improved resolution structure of the human beta common receptor involved in IL-3, IL-5 and GM-CSF signalling which gives better definition of the high-affinity binding epitope. Acta Crystallogr Sect F Struct Biol Cryst Commun.

[46] Miura O, Ihle JN (1993). Dimer- and oligomerization of the erythropoietin receptor by disulfide bond formation and significance of the region near the WSXWS motif in intracellular transport. Arch Biochem Biophys.

[47] Swartjes M (2011). ARA290, a peptide derived from the tertiary structure of erythropoietin, produces long-term relief of neuropathic pain: an experimental study in rats and beta-common receptor knockout mice. Anesthesiology.

[48] Corbett MS, Mark AE, Poger D (2017). Do All X-ray Structures of Protein-Ligand Complexes Represent Functional States? EPOR, a Case Study. Biophys J.

[49] Constantinescu SN (2001). Ligand-independent oligomerization of cell-surface erythropoietin receptor is mediated by the transmembrane domain. Proceedings of the National Academy of Sciences of the United States of America.

[50] PyMOL Molecular Graphics System v. Version 1.5.0.4 (LLC).

[51] Sybyl-X Molecular Modeling Software Packages v. 2.1 (USA, 2013).

[52] Comeau SR, Gatchell DW, Vajda S, Camacho CJ (2004). ClusPro: a fully automated algorithm for protein-protein docking. Nucleic Acids Res.

[53] Kozakov D, Brenke R, Comeau SR, Vajda S (2006). PIPER: an FFT-based protein docking program with pairwise potentials. Proteins.

[54] Krissinel E, Henrick K (2007). Inference of macromolecular assemblies from crystalline state. J Mol Biol.

[55] Deng L (2014). PredHS: a web server for predicting protein-protein interaction hot spots by using structural neighborhood properties. Nucleic Acids Res.

[56] Zhu X, Mitchell JC (2011). KFC2: a knowledge-based hot spot prediction method based on interface solvation, atomic density, and plasticity features. Proteins.

[57] Derijard B (1994). JNK1: a protein kinase stimulated by UV light and Ha-Ras that binds and phosphorylates the c-Jun activation domain. Cell.

[58] Broughton SE (2014). Dual mechanism of interleukin-3 receptor blockade by an anti-cancer antibody. Cell Rep.

[59] Laue, T. M., Shah, B. D., Ridgeway, T. M. & Pelletier, S. L. *Computer-aided interpretation of analytical sedimentation data for proteins*. 90–125 (1992).

[60] Schuck P (2000). Size-distribution analysis of macromolecules by sedimentation velocity ultracentrifugation and lamm equation modeling. Biophys J.

[61] Schuck P, Perugini MA, Gonzales NR, Howlett GJ, Schubert D (2002). Size-distribution analysis of proteins by analytical ultracentrifugation: strategies and application to model systems. Biophys J.

[62] Ilsley, M. D. *et al*. Kruppel-like factors compete for promoters and enhancers to fine-tune transcription. *Nucleic Acids Res*, 10.1093/nar/gkx441 (2017). 10.1093/nar/gkx441 PMC5499887 28541545

