# Author Correction: EPO does not promote interaction between the erythropoietin and beta-common receptors

**DOI:** 10.1038/s41598-019-43988-9

**Published:** 2019-05-21

**Authors:** Karen S. Cheung Tung Shing, Sophie E. Broughton, Tracy L. Nero, Kevin Gillinder, Melissa D. Ilsley, Hayley Ramshaw, Angel F. Lopez, Michael D. W. Griffin, Michael W. Parker, Andrew C. Perkins, Urmi Dhagat

**Affiliations:** 1 ACRF Rational Drug Discovery Centre, St. Vincent’s Institute of Medical Research, Fitzroy, Victoria 3065 Australia; 2 Department of Biochemistry and Molecular Biology, Bio21 Molecular Science and Biotechnology Institute, University of Melbourne, Parkville, Victoria 3010 Australia; 3 Australian Centre for Blood Diseases, Monash University, 99 Commercial Road, Melbourne, Victoria 3004 Australia; 4 Mater Research, University of Queensland and Metro South Health Care, South Brisbane, Queensland 4101 Australia; 5 Centre for Cancer Biology, SA Pathology and the University of South Australia, Adelaide, South Australia 5000 Australia

Correction to: *Scientific Reports* 10.1038/s41598-018-29865-x, published online 20 August 2018

In Figure 1C, Sites 1 and 2 are incorrectly labelled. The correct Figure 1 appears below.

**Figure 1:**
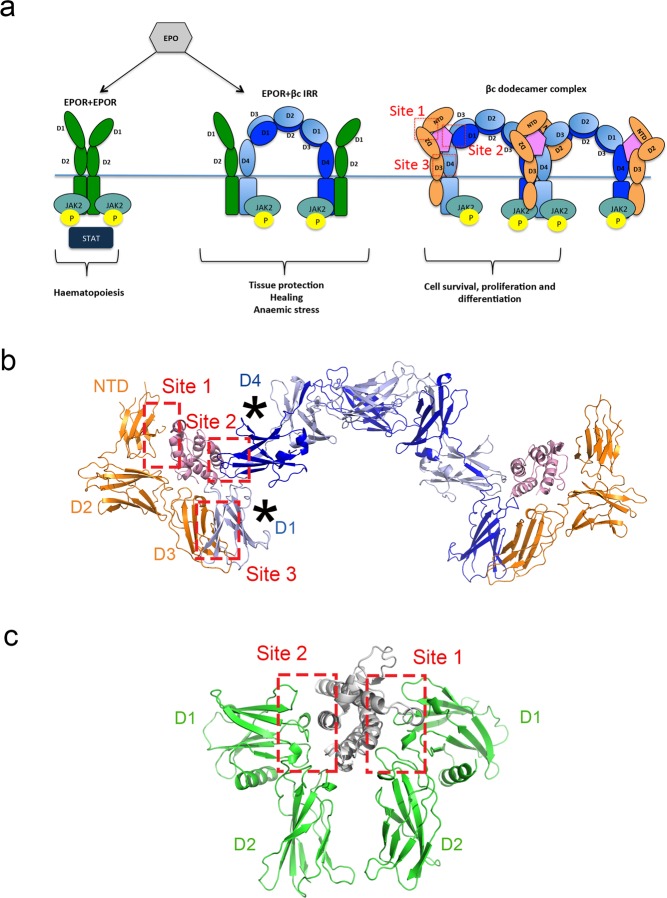
Signalling pathways triggered by EPOR homodimers, the putative EPOR:βc IRR heterodimer and the GM-CSF:GM-CSFRα:βc dodecamer complex. (**a**) EPOR (green) can form homodimers (left) or heterodimers (middle) with the βc receptor (shades of blue). Upon stimulation with EPO (grey), the EPOR homodimer promotes erythroid cell differentiation and survival. The EPOR:βc IRR heterodimer has been hypothesised to promote tissue protection and healing in non-haematopoietic cells and may play a role in anaemic stress. The GM-CSF ternary complex (GM-CSF + GM-CSFRα + βc; shades of pink, orange and blue, respectively) forms higher order signalling complexes (e.g. βc dodecamer complex) and contributes to blood cell survival, proliferation and differentiation. The different sites of interaction in the GM-CSF ternary complex (labelled as βc dodecamer complex) are indicated in red. The cell membrane location is indicated by the horizontal blue line. (**b**) The structure of the GM-CSF:GM-CSFRα:βc hexamer complex (prepared using PDB ID: 4NKQ and 4RS1)^33^ displayed in cartoon format. GM-CSF, GM-CSFRα and βc are coloured pink, orange and shades of blue respectively. For the docking studies, the βc receptor was truncated to the membrane proximal D1/D4 domains only (as indicated by*) due to the size restriction of RosettaDock. The membrane proximal D1/D4 domains are the domains in contact with the cytokine and they participate in the formation of Sites 2 and 3. (**c**) The structure of EPO:EPOR homodimer complex displayed in cartoon format (PDB ID: 1EER)^35^. The location of the EPO:EPOR interaction surfaces, Sites 1 and 2, are indicated. EPO is coloured grey and EPOR green.

