# Supplementary material for: EPO does not promote interaction between the erythropoietin and beta-common receptors
Source: Sci Rep. 2018 Aug 20;8:12457. doi: 10.1038/s41598-018-29865-x (PMC6102255; doi:10.1038/s41598-018-29865-x)
Supplement: Supplementary file 1 — Supplementary Information [file 41598_2018_29865_MOESM1_ESM.pdf]

Supplementary Information

EPO does not promote interaction between the erythropoietin and beta-common receptors.

Karen S. Cheung Tung Shing[1,2], Sophie E. Broughton[1,2], Tracy L. Nero[1,2], Kevin Gillinder[3], Melissa D Ilsley[4], Hayley Ramshaw[5], Angel F. Lopez[5], Michael D. W. Griffin[2], Michael W. Parker[1,2,6], Andrew C. Perkins[3,6] & Urmi Dhagat[1,2,6]

[1]ACRF Rational Drug Discovery Centre, St. Vincent's Institute of Medical Research, Fitzroy, Victoria 3065, Australia

[2]Department of Biochemistry and Molecular Biology, Bio21 Molecular Science and Biotechnology Institute, University of Melbourne, Parkville, Victoria 3010, Australia

[3]Australian Centre for Blood Diseases, Monash University, 99 Commercial Road, Melbourne Victoria 3004, Australia

[4]Mater Research, University of Queensland and Metro South Health Care, South Brisbane, Queensland 4101, Australia

[5]Centre for Cancer Biology, SA Pathology and the University of South Australia, Adelaide, South Australia 5000, Australia

[6]Co-senior authors

Supplementary Methods

Purification of the extracellular region of EPOR

Bacmids and baculoviruses were generated using the Invitrogen Bac-to-Bac™ Baculovirus expression system. EPOR was expressed in High Five cell cultures (2.5×10^6 cells/mL) grown in Sf900 II media and incubated at 28°C, for 49 hr. The cell cultures were then centrifuged and the supernatant with the secreted protein filtered, buffer exchanged (10 mM HEPES, 500 mM NaCl, 15% glycerol pH 7.2) and concentrated using the Sartorius Stedim Sartojet tangential flow through system. The supernatant was then loaded onto a His Trap FF column (GE Healthcare) and eluted over a 30 min gradient with 10 mM HEPES, 500 mM NaCl, 15% glycerol, 500 mM imidazole, pH 7.2. Fractions were further purified using a Superdex 200 16/60 pg column (GE Healthcare) (10 mM HEPES, 150 mM NaCl, 15% glycerol pH 7.2). Protein identity, purity, folding and yield were assessed by visualisation on coomassie stained SDS-PAGE gel, anti-His Western blotting (6xHis tag monoclonal antibody MA1-21315-D680, Thermofisher Scientific), tryptic digest mass spectrometry (Bio21 Institute Mass Spectrometry Informatics Laboratory) and circular dichroism. Circular dichroism (CD) data were analysed using the online server Dichroweb (http://dichroweb.cryst.bbk.ac.uk/html/home.shtml) [1] with the CDSSTR algorithm and SMP180 dataset.

Purification of the extracellular region of the βc receptor

Bacmids and baculoviruses were generated using the Invitrogen Bac-to-Bac™ Baculovirus expression system. Expression was carried out in attached *Sf*21 insect cells, with 10×10^6 cells in Sf900II media allowed to attach and grow in T-flasks for 72 hr at 27°C. The cells were then infected with the generated baculovirus and incubated with the virus for one hr, after which the media and virus were drained and replaced with fresh media before another 72 hr incubation for expression. The media with the secreted protein was then harvested from each T-flask and filtered, buffer exchanged (20 mM phosphate, 500 mM NaCl, 20 mM imidazole pH 7) and concentrated using the Sartorius Stedim Sartojet tangential flow through system. The supernatant was then loaded onto a His Trap HP column (GE Healthcare) and eluted (20 mM phosphate, 500 mM NaCl, 500 mM imidazole, pH 7). The fractions were further purified using a Superdex 200 16/60 pg column (GE Healthcare). The purified protein was validated using the methods detailed above for EPOR, except for the CD study set 4 was used as reference dataset with the Dichroweb analysis.

In silico docking of EPOR to the βc receptor in the absence of EPO

The membrane proximal domain 2 (D2) of EPOR (Fig. 1) from each of the three available crystal structures of the EPOR extracellular regions (PDB IDs: 1CN4, 1EER, 1ERN) [2,3] was aligned to the membrane proximal D3 of the α-subunit in the GM-CSF ternary complex (PDB ID: 4NKQ) [4,5] using PyMOL v1.8.2.2 (http://www.pymol.org) [6]. Each aligned EPOR chain (PDB IDs: 1ERN_A, 1EER_B, 1CN4_A) together with the D1 (first βc monomer) and D4 (second βc monomer) of the βc homodimer from the GM-CSF ternary complex was merged into one PDB file, yielding three crude pre-formed IRR complexes for docking studies after all waters and hydrogens were removed using tools within the modelling software SYBYL-X 2.1.1 (Certara L. P.; http://www.certara.com) [7]. It was not possible to use the full βc homodimer due to size restrictions of the RosettaDock software (Rosetta Suite 3.4; http://rosie.graylab.jhu.edu/) [8-10], therefore only the two interacting domains (D1 from βc monomer 1 and D4 from βc monomer 2) were used in the docking studies. To identify the individual EPOR and βc components used in each IRR heteroreceptor model the following nomenclature was used: [EPOR (PDB ID_chain]_[βc_GM-CSF; D1/D4 only]. The three crude pre-formed IRR complexes were used as input to ClusPro2.0 (http://cluspro.bu.edu/login.php) [11,12] for a blind rigid dock, with the βc (D1/D4 only) defined as the receptor and EPOR (D1/D2) as the ligand. In the blind rigid body dock, the ligand is moved around the receptor without any spatial restriction and the top 2000 solutions (ranked by interaction energy) were clustered by the ligand pose (i.e. EPOR pose). The ligands (i.e. EPOR) of the top 30 cluster centres were geometrically optimised using the CHARMM force field in the presence of the receptor (i.e. βc), to produce the final protein complex models. A biased dock of βc (D1/D4 only) and EPOR was also carried out, where the three crude EPOR:βc (D1/D4 only) models were used as input for RosettaDock. In the biased docking process, the initial guess of the EPOR:βc protein complex underwent a rigid body docking search followed by flexible side-chain optimisation. One thousand independent simulations were then performed and the resulting protein complex models were ranked based on interaction energy. For each of the three biased docking calculations, the top ten ranked models were retained and visually inspected.

D2 of EPOR and D4 of the βc receptor are the membrane proximal domains of each of these receptors (Fig. 1) and they are connected to their respective transmembrane regions by a short juxtamembrane region. During the analysis of the docking results, the assumption was that D2 of EPOR must interact with D4 of βc to form Site 3 (i.e. formation of the IRR, Fig. 1) and any docking solutions not meeting this requirement were discarded. The interface(s) between the βc receptor and EPOR in the modelled IRR complexes were analysed using the Protein interfaces, surfaces and assemblies service (PISA)

at the European Bioinformatics Institute (http://www.ebi.ac.uk/pdbe/prot_int/pistart.html) [13]. The PredHS (http://www.predhs.org/) [14] and KFC2 (https://mitchell-lab.biochem.wisc.edu/KFC_Server/index.php) [15] web servers were used to predict hot spot residues within the protein-protein interaction interface. All docking results were visualised in PyMOL. The details for feasible pre-formed EPOR:βc IRR heteroreceptor models are tabulated in Supplementary Tables S1 and S2.

Supplementary Results

In silico docking of the extracellular domains of EPOR and the βc receptor in the absence of EPO

In the absence of EPO, blind rigid body docking yielded only one solution (Model 1, Supplementary Fig. S1a) in which the receptors were orientated with their membrane proximal domains (D2 of the EPOR and D4 of βc) in alignment. However, the arrangement of EPOR and βc in Model 1 is very different to that observed in the EPOR homodimer or the GM-CSFRα:βc portion of the GM-CSF ternary complex (Fig. 1) and there is little contact between the membrane proximal domains of the two receptors (referred to as Site 3, Supplementary Fig. S1). Thus, Model 1 is an unlikely representation of the extracellular domains of the pre-formed IRR heteroreceptor.

The interactions between residues at Site 3 for Models 2-4 (biased docking models) were analysed using PISA, followed by PredHS and KFC2. These analyses predicted residue E173 from EPOR, as well as N393 and S396 from the βc receptor, to be critical to the Site 3 interface (i.e. hotspot residues, Supplementary Tables S1 and S2). Of these, N393 and H396 from the βc receptor have been reported to mediate interactions between βc and the GM-CSFRα at Site 3 in the GM-CSF ternary complex (PDB ID: 4NKQ) [16].

Supplementary Table S1 Summary of the EPOR interactions at the Site 3 interface with the βc receptor in IRR heteroreceptor models 2-4.

The EPOR and βc (D1/D4 only) components from the available crystal structures used to construct the IRR heteroreceptor models are denoted as [EPOR PDB ID_chain]_[βc _GM-CSF]. The βc (D1/D4 only) was extracted from the GM-CSF ternary complex (PDB ID: 4NKQ) [16]. The residues predicted to form a hydrogen bond and/or salt bridge at the Site 3 interface are denoted by H and S, respectively. Residues present at the Site 3 interface but not forming any hydrogen bond or salt bridge are denoted by I. The buried area percentages are also shown. Residues predicted to be interaction hotspots at the Site 3 interface are shown in orange if predicted by KFC2 only, yellow if predicted by PredHS only and red if predicted by both KFC2 and PredHS.

MODEL	2	3	4
**Crystal structure components**	**[1CN4_A]_[βc_GM-CSF]**	**[1ERN_B]_[βc_GM-CSF]**	**[1EER_B]_[βc_GMCSF]**
**EPOR residue**			
HIS 137		I-20%	
SER 152			I-50%
HIS 153			I-50%
ARG 155	H-90%	I-10%	HS-70%
GLU 157	I-50%		I-50%
ALA 166	I-30%		
GLY 167	I-10%		
SER 168	I-30%	I-20%	I-20%
VAL 169	H-30%	I-20%	I-30%
GLN 170	H-70%	H-60%	I-40%
ARG 171	I-40%	I-50%	H-50%
VAL 172	I-80%	I-90%	I-70%
GLU 173	H-100%	HS-90%	HS-100%
ILE 174		I-20%	I-90%
LEU 175	I-50%	I-50%	H-90%
GLU 176	H-50%	I-50%	S-90%
GLY 177		I-10%	I-20%
ARG 178		HS-80%	HS-50%
GLU 180		I-10%	I-10%
CYS 181		I-70%	I-30%
VAL 182		H-70%	I-30%
LEU 183		I-50%	
SER 184		I-70%	

ASN 185	I-70%	H-60%	H-30%
ARG 187	H-50%		
ARG 199	I-80%		
MET 200	I-20%		
ALA 201	I-60%		I-40%
GLU 202	HS-70%		I-30%
PRO 203	I-20%		I-20%
TRP 209	I-40%		I-20%

Supplementary Table S2 Summary of the βc receptor interactions at the Site 3 interface with EPOR in IRR heteroreceptor models 2-4.

The EPOR and βc (D1/D4 only) components from the available crystal structures used to construct the IRR heteroreceptor models are denoted as [EPOR PDB ID_chain]_[βc _GM-CSF]. The βc (D1/D4 only) was taken from the GM-CSF ternary complex (PDB ID: 4NKQ). Residues predicted to form a hydrogen bond and/or salt bridge at the Site 3 interface are denoted by H and S, respectively. The residues present at the Site 3 interface but not forming any hydrogen bond or salt bridge are denoted by I. The buried area percentages are also shown. Residues predicted to be interaction hotspots at the Site 3 interface are shown in orange if predicted by KFC2 only, yellow if predicted by PredHS only and red if predicted by both KFC2 and PredHS.

MODEL	2	3	4
**Crystal structure components**	**[1CN4_A]_[βc_GM-CSF]**	**[1ERN_B]_[βc_GM-CSF]**	**[1EER_B]_[βc_GMCSF]**
**βc residue**			
ASP 350	I-30%		I-30%
SER 353			I-50%
SER 355	I-20%		I-20%
ARG 357		HS-50%	HS-40%
GLU 359		I-20%	HS-40%
THR 360		I-10%	I-10%
LYS 362		I-30%	S-60%
MET 363		I-20%	
ARG 364		I-10%	I-10%
TYR 365	I-20%		
GLU 366	I-40%	H-60%	I-60%
HIS 367	HS-100%	I-30%	I-10%
ILE 368	I-20%	I-10%	I-20%
ASP 369	I-100%	HS-70%	HS-70%
HIS 370			I-80%
LYS 387	I-10%		I-10%
GLU 389	I-60%	H-40%	H-40%
THR 390	I-10%	I-30%	I-10%
LEU 391	I-80%	I-90%	I-40%
GLN 392	H-40%	I-70%	I-30%
ASN 393	I-80%	H-80%	H-100%
ALA 394	I-100%	I-70%	I-90%

HIS 395	I-10%	I-70%	H-100%
SER 396	H-70%	I-80%	H-100%
MET 397	I-80%	I-40%	
ALA 398	H-50%	H-60%	
LEU 399	I-40%		
PRO 400	I-30%		
SER 417	I-20%		
ARG 418	H-70%	I-10%	I-10%
THR 419	H-40%		
TYR 421	I-10%		

In silico docking of the extracellular domains of EPOR and the βc receptor in the presence of EPO

The models generated using RosettaDock in scenario one were analysed by PISA, followed by PredHS and KFC2. These analyses predicted residues E13 and E18 from EPO and S91, F93, V94, N116, H153, P203, S204 and F205 from EPOR to be hotspot residues involved at Site 1 (Models 5-9 Supplementary Tables S3-S6). Some of these predicted EPOR hotspot residues have been reported to be key interacting residues in the EPO:EPOR homodimer structure; EPOR residues F93, V94, N116, H153 and S204 in Site 1 and F93, V94 and S204 in Site 2 [2]. βc receptor residues R64 and V105 were also predicted to be hotspot residues at the EPO:βc interface (Site 2) in Models 5-9 (Supplementary Tables S3- S6).

Supplementary Table S3 Summary of the EPO interactions with EPOR and the βc receptor in models 5-9.

The EPO:EPOR:βc IRR heteroreceptor was modelled as described in the Materials and methods and illustrated in Fig. 4. The EPO residues predicted to form a hydrogen bond and/or salt bridge at Site 1 with EPOR, or Site 2 with the βc receptor, are denoted by H and S, respectively. EPO residues present at the Site 1 or 2 interface but not forming any hydrogen bond or salt bridge are denoted by I. The buried area percentages are also shown. Residues predicted to be interaction hotspots at the Site 1 or 2 interfaces are shown in orange if predicted by KFC2 only, yellow if predicted by PredHS only and red if predicted by both KFC2 and PredHS. Site 1 and 2 interactions are shown in black and blue text, respectively.

Model	5	6	7	8	9
EPO residue					
ALA 1					I-10%
PRO 2					I-20%
ARG 4	H-20%	I-10%			
LEU 5	I-90%	I-70%	I-40%		
ASP 8	S-70%	S-70%	HS 60%		I-10%
SER 9	I-30%+I-20%	I-30%+I-20%	I-30% +I-10%	I-70%	I-60%
ARG 10	I-10%+I-60%	I-10%+HS-60%	I-10%+HS-70%	HS-20%+I-30%	I-20%+I-20%
VAL 11	I-20%+I-70%	I-20%+ I-50%	I-10%+I-40%		I-40%
GLU 13	I-30%	I-30%+I60%	I-30%	I-70% +I-20%	I-30%+I-30%
ARG 14	I-70%+I-40%	I-90%+I-50%	I-60%+I-40%	I-10%	HS-100%
TYR 15	I-90%+I-10%		I-80%		I-80%
LEU 16	I-100%	I-100%	I-100%	I-80%	
LEU 17	I-30%+I-10%	I-30%+I-20%	I-30%+I-10%	I-40%+I-60%	I-60%
GLU 18	I-80%	I-80%	I-70%	I-20%	I-80%
LYS 20	I-30%	I-30%	I-30%	I-30%	
GLU 21				I-60%	I-70%
GLU 23	I-20%	I-20%	I-20%		
LYS 24				I-20%	I-10%
ILE 25					I-10%
ASP 43	I-20%	I-20%	I-20%		
THR 44	I-80%	I-80%	I-80%	I-10%	
LYS 45	S-60%	S-60%	S-60%	S-50%	
VAL 46	H-100%	H-100%	H-100%	I-100%	
ASN 47	H-80%	H-80%	H-80%	I-40%	
PHE 48	I-100%	I-100%	I-100%	I-100%	
TYR 49	I-40%	I-40%	I-40%	I-30%	

LYS 52	I-20%	I-20%	I-20%	HS-30%	
GLU 89	I-40%	I-50%	I-50%	I-40%	
PRO 90		I-20%	I-50%	I-40%	
GLN 92	I-20%	I-30%	I-30%		
LEU 93	I-70%	I-70%+I-80%	I-70% +I-80%	I-30%	I-10%
ASP 96	I-30%	I-20%	I20%		
LYS 97	I-50%	I-60%	I-60%	I-10%	I-60%
SER 100	I-20%+I-30%	I-10%+I-10%	I-20%		I-60%
GLY 101	I-100%	I-100%	I-100%		I-80%
ARG 103					HS-30%
SER 104	I-10%				I-100%
THR 107					I-30%
LEU 108					I-10%
ARG 131				I-10%	
ILE 133	I-70%	I-70%	I-70%		
THR 134	I-10%	I-10%	I-10%		
LYS 140	HS-50%	HS-50%	HS-50%		
ARG 143	I-40%	I-40%	I-40%	HS-40%	
VAL 144	I-50% + I-30%	I-50%	I-50%		
ASN 147	I-100%	I-100%	I-100%	H-90%	
ARG 150	HS-90%	HS-90%	HS-90%	H-90%	
GLY 151	I-100%	I-100%	I-100%	I-90%	
LYS 154	I-70%	I-70%	I-70%	I-90%	I-20%
LEU 155	I-80%	I-80%	I-80%	I-60%	
GLY 158	I-30%	I-30%	I-30%	I-40%	
GLU 159					I-10%
CYS 161					I-20%
ARG 162	I-20%	I-20%	I-20%	I-30%	H-80%
THR 163					I-20%
GLY 164					H-70%
ASP 165					I-30%
ARG 166					H-20%

Supplementary Table S4 Summary of the EPOR interactions in the EPO:EPOR:βc IRR heteroreceptor models 5-9.

The EPO:EPOR:βc IRR heteroreceptor was modelled as described in the Materials and methods and illustrated in Fig. 4. The EPOR residues predicted to form a hydrogen bond and/or salt bridge at Site 1 (with EPO) or Site 3 (with βc) are denoted by H and S, respectively. EPOR residues present at the Site 1 or 3 interface but not forming any hydrogen bond or salt bridge are denoted by I. The buried area percentages are also shown. Residues predicted to be interaction hot spots at the Site 1 or 3 interfaces are shown in orange if predicted by KFC2 only, yellow if predicted by PredHS only and red if predicted by both KFC2 and PredHS. Site 1 and 3 interactions are shown in black and green text, respectively.

Model	5	6	7	8	9
**EPOR residue**					
ARG 32	I-10%	I-10%	I-10%		
LEU 33	I-70%	I-70%	I-70%	I-60%	
GLU 34	I-50%	I-50%	I-50%	HS-40%	I-10%
LEU 59	I-50%	I-50%	I-50%	I-50%	
GLU 60	I-50%	I-50%	I-50%	HS-60%	I-50%
ASP 61	HS-50%	HS-50%	HS-50%	HS-20%	I-50%
GLU 62	S-40%	S-40%	S-40%	S-40%	HS-40%
THR 87	H-50%	H-50%	H-50%	I-60%	I-10%
ALA 88	H-20%	H-20%	H-20%	I-70%	
ASP 89				I-20%	
THR 90	I-90%	I-90%	I-90%	I-100%	
SER 91	H-90%	H-90%	H-90%	I-100%	I-50%
SER 92	H-100%	H-100%	H-100%	I-100%	I-80%
PHE 93	I-100%	I-100%	I-100%	I-90%	I-100%
VAL 94	I-80%	I-80%	I-80%	I-60%	I-90%
PRO 95	I-80%	I-80%	I-80%	I-10%	I-40%
VAL 112	I-10%	I-10%	I-10%		
HIS 114	I-60%	I-60%	I-60%		I-50%
ASN 116	H-100%	H-100%	H-100%	I-80%	I-100%
GLU 117	HS-30%	HS-30%	HS-30%	I-20%	HS-30%
THR 148				I-20%	
PRO 149	I-10%	I-10%	I-10%	I-30%	
MET 150	I-70%	I-70%	I--70%	I-90%	I-40%
THR 151	I-10%	I-10%	I-10%	I-30%	
SER 152	I-10%	I-10%	I-10%	I-20%	I-10%
HIS 153	I-60%	I-60%	I-60%	I-90%	I-20%
ARG 155				I-100%	I-90%
GLU 157				I-50%	I-50%
ALA 166				I-30%	I-30%

GLY 167				I-10%	I-10%
SER 168				I-30%	I-30%
VAL 169				I-30%	I-30%
GLN 170				H-80%	H-70%
ARG 171				I-40%	I-40%
VAL 172				I-80%	I-80%
GLU 173				H-100%	H-100%
LEU 175	I-10%	I-10%+I-10%	I-10%	I-50%	I-50%
GLU 176	I-10%	I-10%	I-10%	HS-40% +I-40%	I-40%
ASN 185				H-80%	H-80%
ARG 187				H-60%	H-60%
ARG 199				I-60%	I-70%
MET 200				I-20%	I-20%
ALA 201				I-10%+I-60%	I-60%
GLU 202	I-30%	I-30%	I-30%	I-10%+HS-70%	H-70%+I 10%
PRO 203	H-90%	H-90%	H-90%	I-50%+I-30%	I-20%+H-50%
SER 204	H-90%	H-90%	H-90%	H-90%	I-80%
PHE 205	I-70%	I-70%	I-70%	I-60%	I-60%
TRP 209				I-30%	I-30%

Supplementary Table S5 Summary of the βc D1 interactions with EPO and EPOR in the EPO:EPOR:βc IRR heteroreceptor models 5-9.

The EPO:EPOR:βc IRR heteroreceptor was modelled as described in the Materials and methods and illustrated in Fig. 4. The residues predicted to form a hydrogen bond and/or salt bridge at Site 2 with EPO or Site 3 with EPOR are denoted by H and S, respectively. βc D1 residues present at the Site 2 or 3 interfaces but not forming any hydrogen bond or salt bridge are denoted by I. The buried area percentages are also shown. Residues predicted to be interaction hotspots at the Site 2 or 3 interfaces are shown in orange if predicted by KFC2 only, yellow if predicted by PredHS only and red if predicted by both KFC2 and PredHS. Site 2 and 3 interactions are shown in blue and green text, respectively.

Model	5	6	7	8	9
**βc D1 residue**					
TYR 39	I-10%	I-10%	I-10%		
ARG 64	I-20%	I-20%	I-20%	I-10%	I-20%
VAL 65	I-20%	I-20%	I-20%		H-30%
ASN 66	I-20%	I-30%	I-50%	I-10%	I-100%
GLU 67	I-50%	I-60%	I-70%	I-50%	H-30%
ASP 68	I-20%	I-30%	I-30%	I-20%	H-70%
LEU 69					I-40%
LEU 70					I-10%
SER 102					I-10%
PHE 103	I-20%	I-20%	I-20%		I-10%
VAL 104	I-60%	I-70%	I-70%	I-30%	I-60%
VAL 105	I-50%	I-50%	I-30%	I-30%+I-30%	I-10%+I-20%
THR 106	I-100%	I-100%	I-90%	I-70%	I-60%
ASP 107	I-20%	I-20%	I-20%		I-10%
VAL 108	I-10%	I-10%	I-10%	I-10%	H-20%
TYR 110	I-10%	I-30%	I-30%	I-20%	I-20%

Supplementary Table S6 Summary of the βc D4 interactions with EPO and EPOR in the EPO:EPOR:βc IRR heteroreceptor models 5-9.

The EPO:EPOR:βc IRR heteroreceptor was modelled as described in the Materials and methods and illustrated in Fig. 4. The residues predicted to form a hydrogen bond and/or salt bridge at Site 2 with EPO or Site 3 with EPOR are denoted by H and S, respectively. βc D4 residues present at the Site 2 or 3 interfaces but not forming any hydrogen bond or salt bridge are denoted by I. The buried area percentages are also shown. Residues predicted to be interaction hotspots at the Site 2 or 3 interfaces are shown in orange if predicted by KFC2 only, yellow if predicted by PredHS only and red if predicted by both KFC2 and PredHS. Site 2 and 3 interactions are shown in blue and green text, respectively.

Model	5	6	7	8	9
**βc D4 residue**					
LYS 333	I-20%	I-10%		I-10%	
ASP 350				I-20%	I-20%
SER 355				I-10%	I-10%
ARG 364	I-10%	I-10%	H-10%		
TYR 365	I-50%	I-50%	I-50%	I-10%	I-10%+I-20%
GLU 366	I-20%	HS20%+I-10%	HS-20%	I-40%	I-40%
HIS 367	I-70%	S-70%	I-60%	S-100%	H-100%
ILE 368	I-10%			I-10%	I-10%
ASP 369				I-100%	I-100%
GLU 389				H-10%	H-40%
THR 390				I-10%	I-10%
LEU 391				I-70%	I-70%
GLN 392				H-40%	H-40%
ASN 393				I-80%	I-80%
ALA 394				I-100%	I-100%
HIS 395				I-10%	I-10%
SER 396				H-60%	H-60%
MET 397				I-70%	I-70%
ALA 398				H-50%	H-50%
LEU 399				I-40%	I-40%
PRO 400				I-30%	I-30%
SER 417				I-10%	I-20%
ARG 418	S-70%	S-60%	HS-80%	H-80%+ I-20%	H-80%
THR 419	H-40%	I-20%	I-20%	I-40%+I-30%	I-40%
GLY 420	I-40%	I-20%	I-10%	I-20%	
TYR 421	I-40%	I-40%	I-20%	I-20%	I-10%

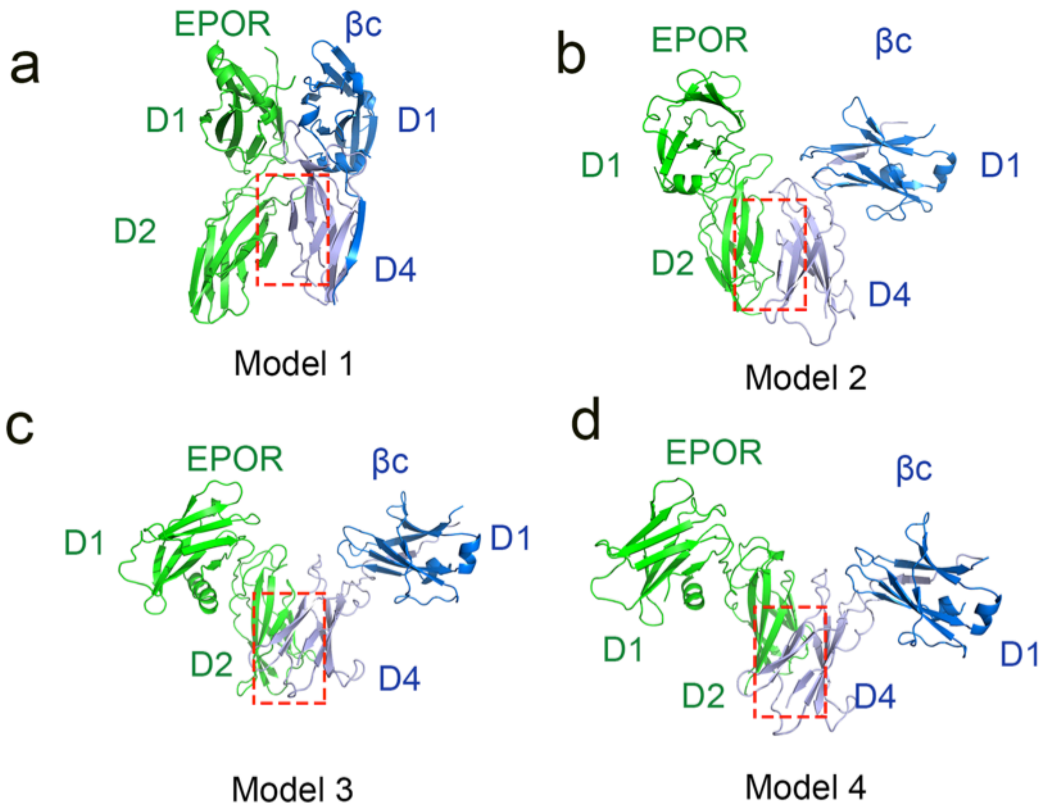

Supplementary Fig. S1 Docking the extracellular domains of EPOR and the βc receptor in the absence of EPO.

In the absence of EPO, the membrane proximal domains of EPOR (i.e. D2) and βc (i.e. D4) can interact to form Site 3. The extracellular domain of EPOR is shown as a green cartoon and βc (D1/D4 only) in shades of blue. Pre-formed EPOR:βc IRR heteroreceptor models obtained from (a) blind rigid body docking using ClusPro2.0 (Model 1) and (b-d) biased docking using RosettaDock (Models 2-4), with Site 3 indicated by the red box.

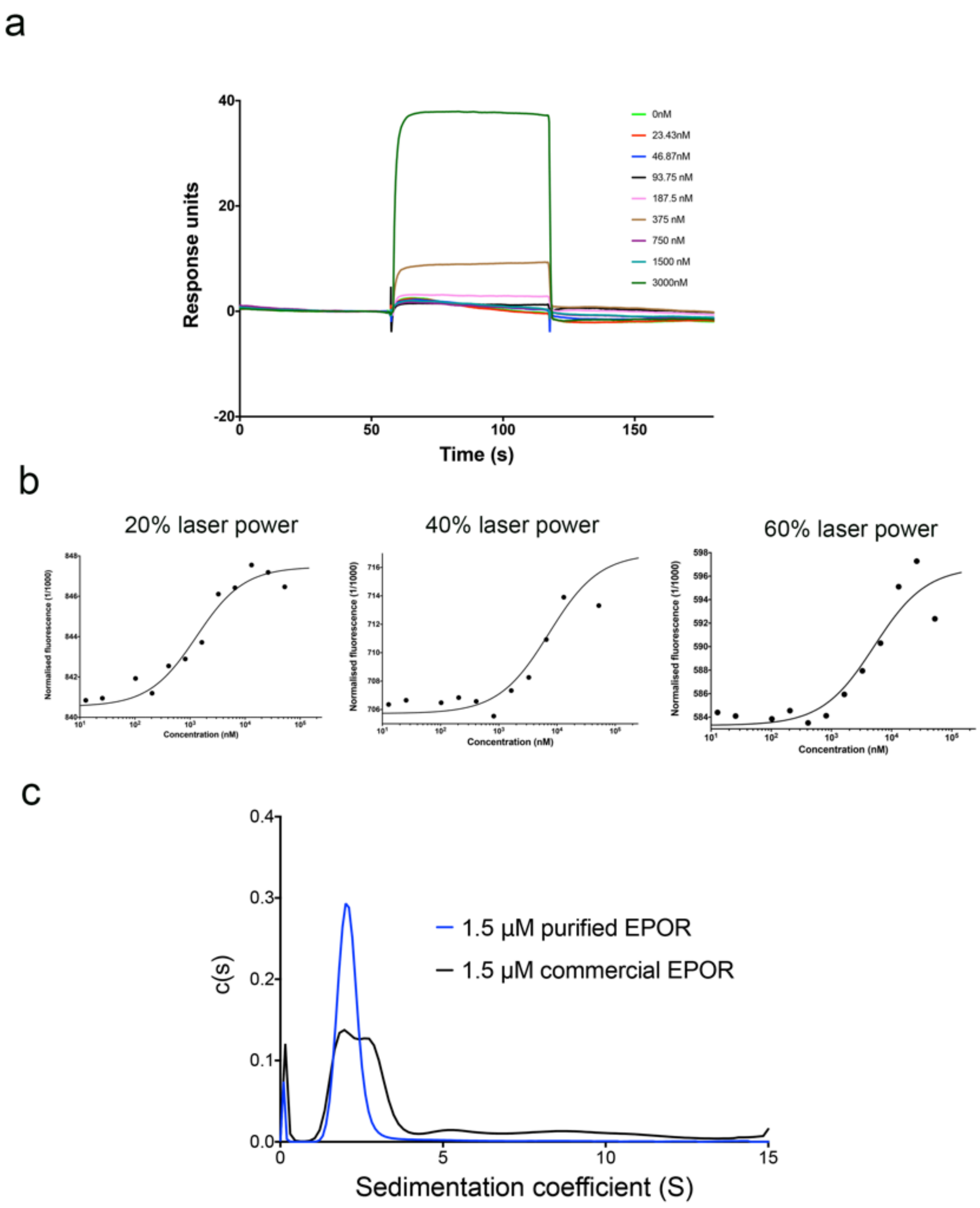

Supplementary Fig. S2 The commercially acquired EPOR was aggregated and showed non-specific binding.

The commercial EPOR showed non-specific binding in (a) SPR and (b) MST experiments carried out using the same methodology as for the in-house produced EPOR protein. (c) AUC indicated that the commercial EPOR was aggregated, with several peaks at different sedimentation coefficients and thus the material was deemed unsuitable for the biophysical experiments, while the in-house purified EPOR was properly folded and homogeneous.

a

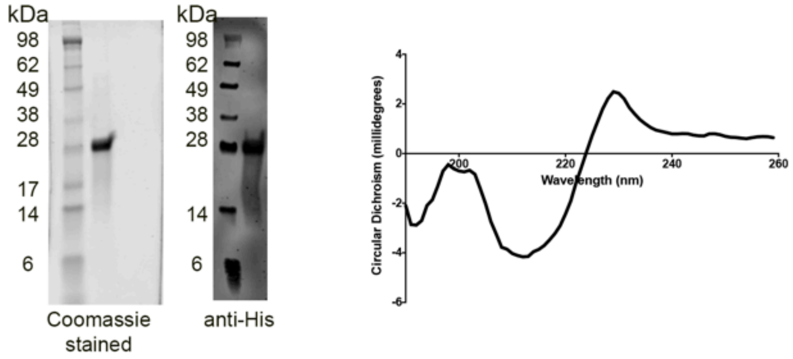

>EPOR_HUMAN
Matched peptides shown in red

MDHLGASLWPQVGSLCLLLAGAAWAPPPNLPDPKFESKAALLAARGPEELLCF
TERLEDLVCFWEEAASAGVGPGNYSFSYQLEDEPWKLCRLHQAPTARGAVRF
WCSLPTADTSSFVPLELRVTAASGAPRYHRVIHINEVVLLDAPVGLVARLADES
GHVVLRWLPPPETPMTSHIRYEVDVSAGNGAGSVQRVEILEGRTECVLSNLRG
RTRYTFAVRARMAEPSFGGFWSAWSEPVSLLTPSDLD

b

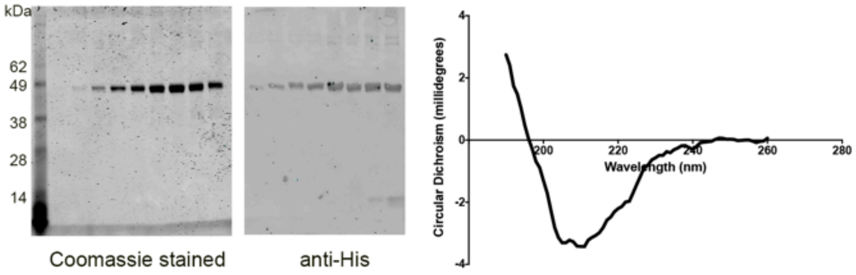

>Beta common subunit (Human)
Matched peptides shown in red

EETIPLQTLRCYNDYTSHITCRWADTQDAQRLVNVTLIRRVNEDLLEPVSCDLSD
DMPWSACPHPRCVPRRCVIPCQSFVVTDVDYFSFQPDRPLGTRLTVTLTQHVP
PEPRDLQISTDQDHFLLTWSVALGSPQSHWLSPGDLEFEVVYKRLQDSWEDAA
ILLSNTSQATLGPEHLMPSSTYVARVRTRLAPGSRLSGRPSKWSPEVCWDSPG
DEAQPQNLECFFDGAAVLSCSWEVRKEVASSVSFGLFYKPSPDAGEEECPLRE
GLGSLHTRHHCQIPVPDPATHGQYIVSVQPRRAEKHIKSSVNIQMAPPSLNVTG
DSYSLRWETMKMRYEHIDHTFEIQYRKDTATWKDSKTETLQNAHSMALPALES
TRYWARVRVRTSRTGYNGIWSEWSEARSWDTES

Supplementary Fig. S3 Purification and quality control of the purified EPOR and βc receptor proteins.

The EPOR (a) and βc (b) proteins were purified and visualised on SDS-PAGE gels and anti-His Western blots. The protein bands were digested by trypsin and the digested peptides analysed by mass spectrometry. Circular dichroism analyses indicated correct folding of the proteins

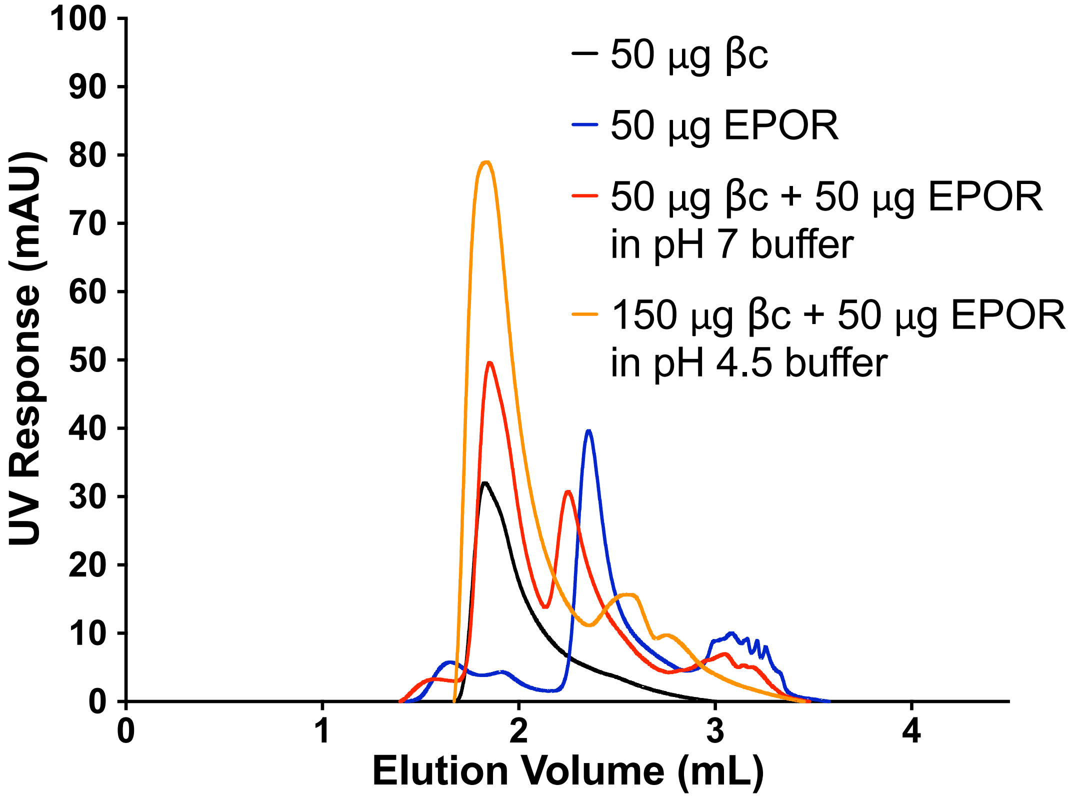

Supplementary Fig. S4 Analytical SEC of purified EPOR and βc receptor proteins.

Overlay of the 280 nm UV absorbance spectra for EPOR, the βc receptor and EPOR+βc (1:1 and 1:3 ratio) injected into the SEC column in two different buffers.

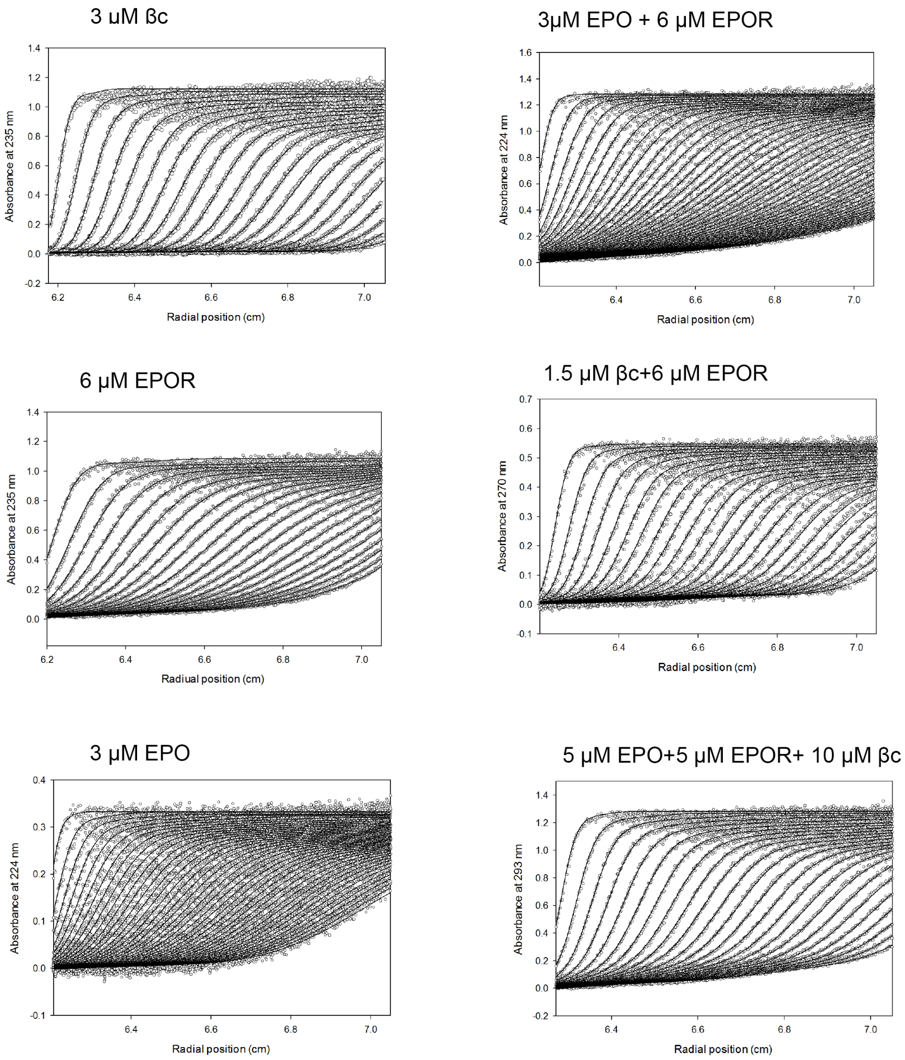

Supplementary Fig. S5 Representative AUC data and curve fits used for calculating c(s) and sedimentation coefficients (S).

The AUC data is shown as round dots and the curve fitting (using Sedfit) as solid lines. The time interval between scans was 10 mins for the 3 µM βc and 6µM EPOR samples and 4 mins for 3 µM EPO, 6 µM EPO+EPOR, 1.5 µM βc+6 µM EPOR and 5 µM EPO+ 5 µM EPOR+ 10 µM βc samples.

A

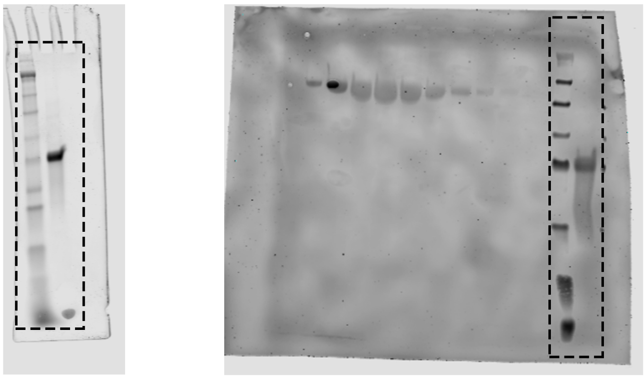

B

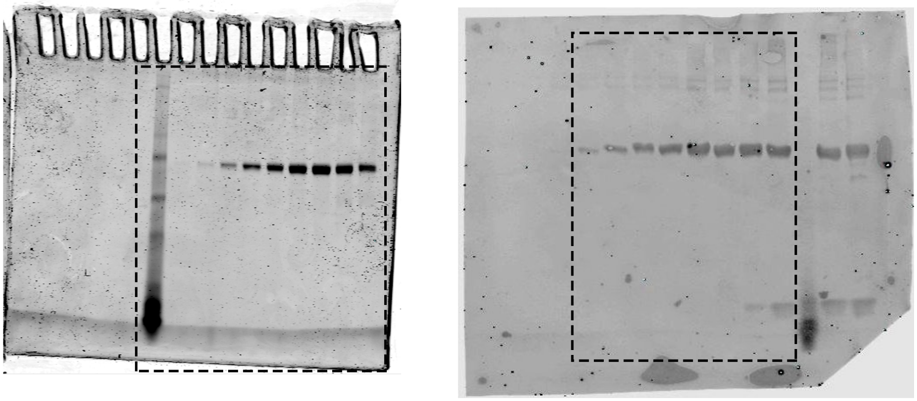

Supplementary Fig. S6 Gels used for generating supplementary Fig. S3

(A) Coomassie stained SDS-PAGE gel (left) and anti-His Western blotting (right) from EPOR-His purification. The areas cropped and used for Supplementary Fig. S3 are shown in the dotted boxes. There was another protein being loaded in parallel on the same Western blotting on the left of the EPOR-His sample. (B) Coomassie stained SDS-PAGE gel (left) and anti-His Western blotting (right) from the βc-His protein purification. The areas cropped for making Supplementary Fig. S3 are shown by dotted boxes.

References

1 Whitmore, L. & Wallace, B. A. DICHROWEB, an online server for protein secondary structure analyses from circular dichroism spectroscopic data. *Nucleic acids research* **32**, W668-673, doi:10.1093/nar/gkh371 (2004).

2 Syed, R. S. *et al.* Efficiency of signalling through cytokine receptors depends critically on receptor orientation. *Nature* **395**, 511-516, doi:10.1038/26773 (1998).

3 Livnah, O. *et al.* Crystallographic evidence for preformed dimers of erythropoietin receptor before ligand activation. *Science* **283**, 987-990 (1999).

4 Broughton, S. E. *et al.* The betac receptor family - Structural insights and their functional implications. *Cytokine* **74**, 247-258, doi:10.1016/j.cyto.2015.02.005 (2015).

5 Hansen, G. *et al.* The structure of the GM-CSF receptor complex reveals a distinct mode of cytokine receptor activation. *Cell* **134**, 496-507, doi:10.1016/j.cell.2008.05.053 (2008).

6 PyMOL Molecular Graphics System v. Version 1.5.0.4 (LLC).

7 Sybyl-X Molecular Modeling Software Packages v. 2.1 (USA, 2013).

8 Lyskov, S. *et al.* Serverification of molecular modeling applications: the Rosetta Online Server that Includes Everyone (ROSIE). *PloS one* **8**, e63906, doi:10.1371/journal.pone.0063906 (2013).

9 Chaudhury, S. *et al.* Benchmarking and analysis of protein docking performance in Rosetta v3.2. *PloS one* **6**, e22477, doi:10.1371/journal.pone.0022477 (2011).

10 Lyskov, S. & Gray, J. J. The RosettaDock server for local protein-protein docking. *Nucleic acids research* **36**, W233-238, doi:10.1093/nar/gkn216 (2008).

11 Comeau, S. R., Gatchell, D. W., Vajda, S. & Camacho, C. J. ClusPro: a fully automated algorithm for protein-protein docking. *Nucleic acids research* **32**, W96-99, doi:10.1093/nar/gkh354 (2004).

12 Kozakov, D., Brenke, R., Comeau, S. R. & Vajda, S. PIPER: an FFT-based protein docking program with pairwise potentials. *Proteins* **65**, 392-406, doi:10.1002/prot.21117 (2006).

13 Krissinel, E. & Henrick, K. Inference of macromolecular assemblies from crystalline state. *Journal of molecular biology* **372**, 774-797, doi:10.1016/j.jmb.2007.05.022 (2007).

14 Deng, L. *et al.* PredHS: a web server for predicting protein-protein interaction hot spots by using structural neighborhood properties. *Nucleic acids research* **42**, W290-295, doi:10.1093/nar/gku437 (2014).

15 Zhu, X. & Mitchell, J. C. KFC2: a knowledge-based hot spot prediction method based on interface solvation, atomic density, and plasticity features. *Proteins* **79**, 2671-2683, doi:10.1002/prot.23094 (2011).

16 Broughton, S. E. *et al*. Conformational Changes in the GM-CSF Receptor Suggest a Molecular Mechanism for Affinity Conversion and Receptor Signaling. *Structure* **24**, 1271-1281, doi:10.1016/j.str.2016.05.017 (2016).
